# Supplementary material for: Highly parallel optimisation of chemical reactions through automation and machine intelligence
Source: Nat Commun. 2025 Jul 12;16:6464. doi: 10.1038/s41467-025-61803-0 (PMC12255721; doi:10.1038/s41467-025-61803-0)
Supplement: Supplementary file 1 — Supplementary Information [file 41467_2025_61803_MOESM1_ESM.pdf]

Supplementary Information

Highly Parallel Optimisation of Chemical Reactions through Automation and Machine Intelligence

Joshua W. Sin, Siu Lun Chau, Ryan P. Burwood,
Kurt Püntener, Raphael Bigler, Philippe Schwaller

Contents

1 Emulator virtual benchmark datasets **2**

2 Baseline benchmarks **4**

3 Noisy benchmarks **12**

4 Constrained benchmarks **13**

5 Experimental section **16**
5.1 HTE platform 16
5.2 Nickel-catalysed Suzuki-Miyaura coupling 17
5.2.1 Reaction condition search space 17
5.2.2 Experimental procedure for HTE campaign with ML optimisation workflow . . 17
5.2.3 Experimental procedure for HTE campaign with experimentalist-designed HTE plates 18
5.2.4 Scale-up of HTE results: Nickel catalysis 20
5.2.5 Scale-up of HTE results: Palladium catalysis 22
5.2.6 Visualisation of experimental results 24
5.2.7 Data analysis of experimental results 24
5.3 Nickel-catalysed Suzuki-Miyaura coupling: Active pharmaceutical ingredient (API) case study 28
5.3.1 Reaction condition search space 28
5.3.2 Experimental procedure for HTE campaign with ML optimisation workflow . . 28
5.3.3 Scale-up of HTE results 28
5.4 Palladium-catalysed Buchwald-Hartwig coupling: API case study 29
5.4.1 Reaction condition search space 29
5.4.2 Experimental procedure for HTE campaign with ML optimisation workflow . . 30
5.4.3 Scale-up of HTE results 30

1 Emulator virtual benchmark datasets

We detail in this section reaction parameters, dataset sizes, and dataset dimensions for all virtual (emulated) benchmark datasets described in the main text (see main text Benchmarking and evaluating section for descriptions of how emulated virtual datasets are constructed). The C-H arylation virtual dataset (Supplementary Figure 1a) was obtained from training a Multi-layer perceptron (MLP) on the experimental data collected from Torres et al.[1] (EDBO+). The Suzuki coupling (suzuki_i - suzuki_iv) multi-objective virtual datasets (Supplementary Figure 1b) were obtained from Olympus [2]. The virtual datasets encompass all possible combinations of the described reaction parameters, created through the multiplicative combinatorial product. The dataset dimension for the C-H arylation dataset is much higher than the Suzuki Coupling datasets due to Density functional theory (DFT) featurisation of the categorical ligand, base, and solvent parameters. The full virtual benchmark datasets are made available as comma separated values (csv) files in the GitHub repository accompanying this paper.

a Pd-catalysed C-H arylation benchmark dataset generated with data from Torres at al. in EDBO+

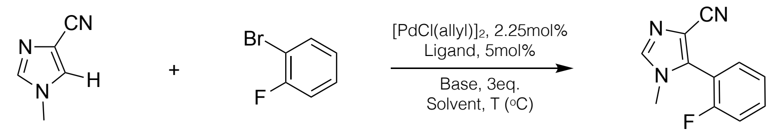

Dataset dimension	Number of reactions
530	7680

Parameter	Unique values	Encoding
ligand	12	DFT
base	4	DFT
solvent	4	DFT
temperature	8	Numerical
concentration	5	Numerical

b Suzuki Coupling suzuki_i - suzuki_iv benchmark datasets from Olympus

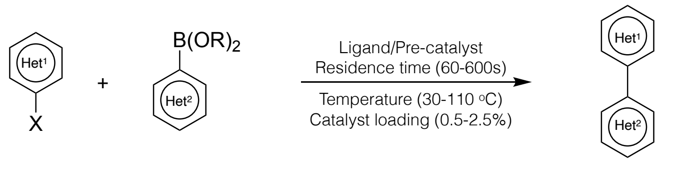

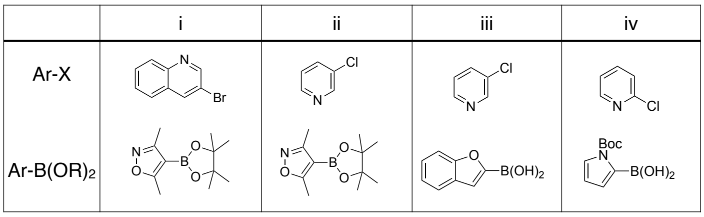

Parameter	Unique values	Encoding
ligand	7	One-hot encoding
residence time	10	Numerical
temperature	9	Numerical
catalyst loading	9	Numerical

Dataset dimension	Number of reactions
10	5670

Supplementary Figure 1: Virtual benchmark datasets used for assessing the performance of optimisation algorithms. Dataset dimension refers to the dimensionality of the featurised reaction representation. The reaction conditions in the virtual datasets encompass all possible combinations of the reaction parameter (ligand, temperature, etc.) values. a, The C-H arylation virtual dataset is generated by training a machine learning (ML) model on 1728 experimentally collected reactions from Torres et al. [1] (EDBO+), then predicting reaction outcomes for a larger range of reaction conditions and variables not present in the original training data. **b**, Four Suzuki coupling virtual datasets from Olympus [2], derived from experimental data, used for benchmarking in this study.

Supplementary Figure 2 shows the distribution of reaction objective values for each virtual benchmark

dataset, highlighting the Pareto optimal points and the Pareto front. Pareto optimal points represent reaction conditions where improving one objective would necessarily lead to a decline in at least one other objective. The Pareto front is formed by connecting these non-dominated points in the objective space. A point is considered non-dominated if no other point is better in all objectives simultaneously. The Pareto front represent a set of optimal trade-offs between multiple objectives [3]. The optimisation algorithms are evaluated on their efficiency in navigating and identifying the best reaction conditions in these benchmark datasets. Their performance is quantified using the hypervolume metric, which measures the volume of objective space enclosed by the Pareto points identified by the optimisation algorithm. The hypervolume (%) is calculated as the ratio of this volume to the volume enclosed by the ground truth Pareto optimal points in the benchmark dataset. We used all datasets except for the Suzuki Coupling (ii) (suzuki_ii) dataset for benchmarking, due to its limited range of yield values (%) and catalyst turnover numbers.

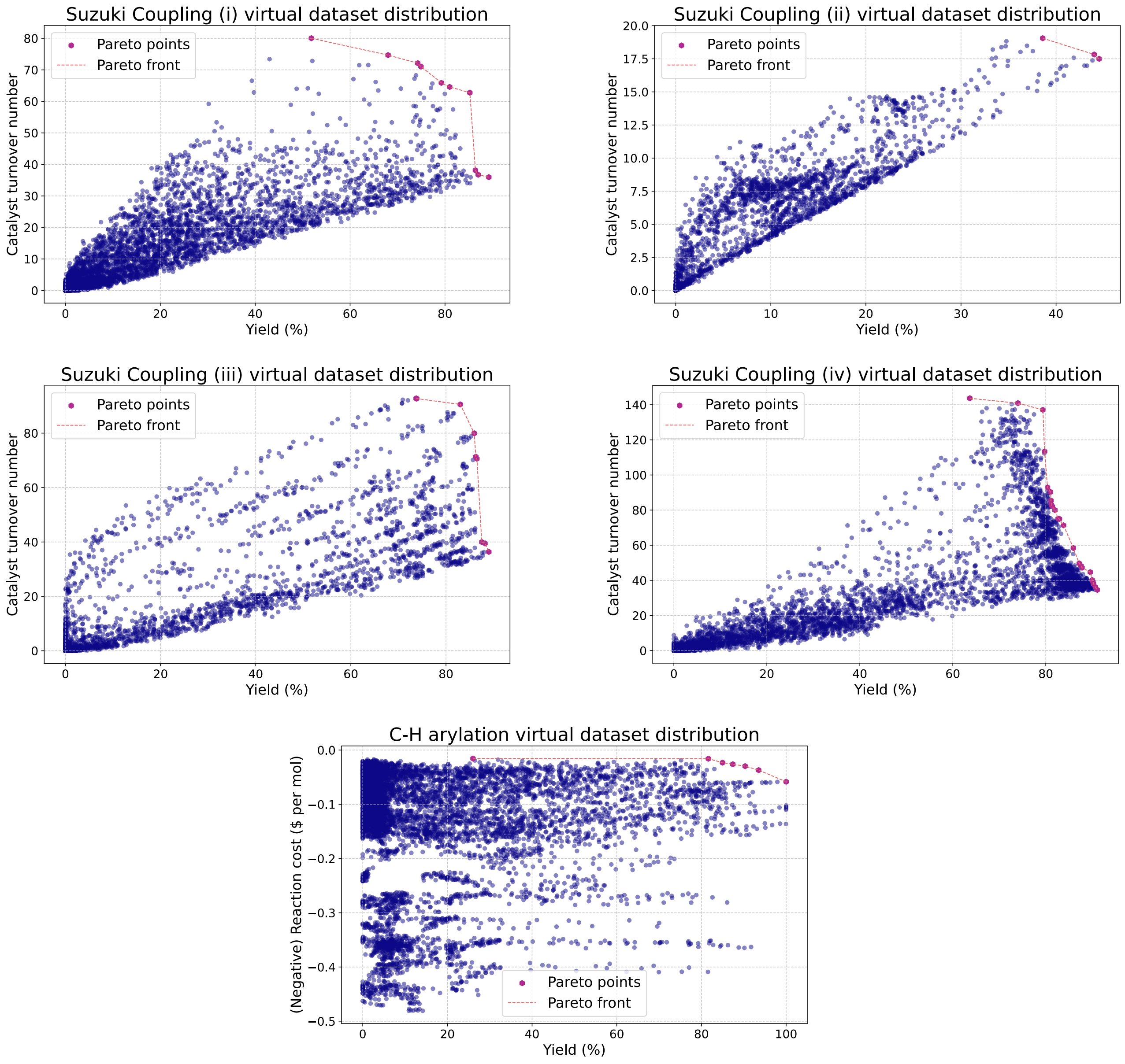

Supplementary Figure 2: Objective distribution of all benchmark datasets. The Pareto optimal points correspond to the reaction conditions with optimal multi-objective trade offs. They are connected to form the Pareto front.

2 Baseline benchmarks

We focused our analysis in the main text on optimisation benchmarks with the lowest batch setting of batch size 24 for 5 iterations (120 total experiments) as most acquisition functions were observed to converge to the ground truth solutions within this budget. This section contains all the optimisation results on benchmark datasets with batch size 24, 48, and 96, for 5 total iterations (Supplementary Figure 3, 4, and 5). As described in the main text (see Optimisation pipeline section), the experiments in the first iteration were selected using quasi-random Sobol sampling from the reaction search space, and the 4 remaining iterations with the Bayesian optimisation algorithm. These experiments were repeated 20 times with different random seeds. Plots of the mean hypervolume % values and the standard deviations are included in this section. The q-NEHVI, qNParEgo, and TS-HVI acquisition functions demonstrate scalability to high batch sizes, even with large high dimensional search spaces as in the C-H arylation benchmark dataset [1]. The tested algorithms also outperform a quasi-random Sobol baseline for all datasets and batch sizes. Supplementary Figure 6 contains statistical significance plots comparing the performance of the different acquisition functions on the benchmark datasets with batch size 24 for 5 iterations. qNEHVI was observed to have the overall best performance across all benchmark datasets, supported by the statistical tests.

Benchmarks with batch size of 24 for 5 iterations (total budget of 120 experiments)

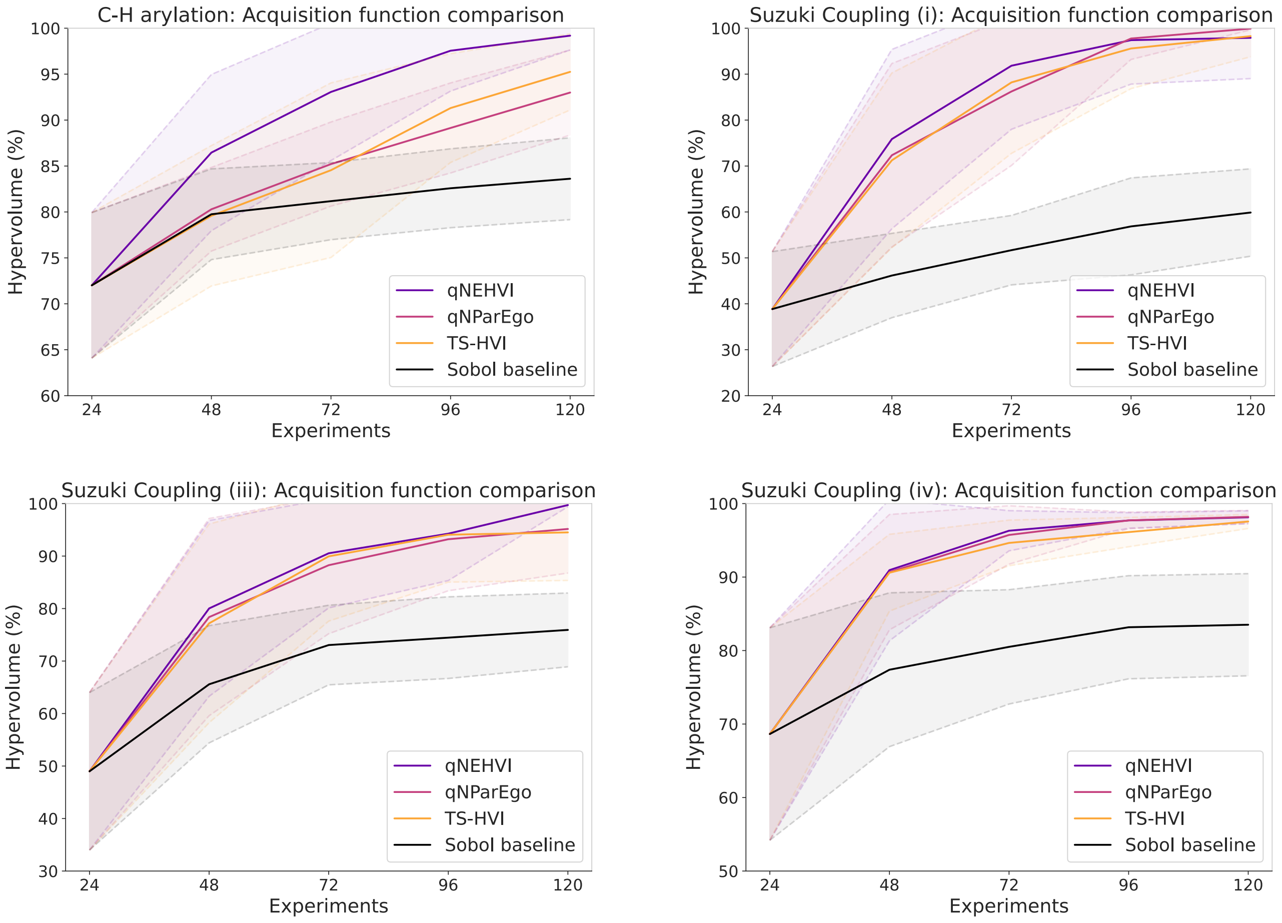

Supplementary Figure 3: Optimisation algorithm performance with batch size 24 on all benchmark datasets. Repeated across 20 different random seeds with plotted hypervolume (%) mean and ± 1 standard deviation.

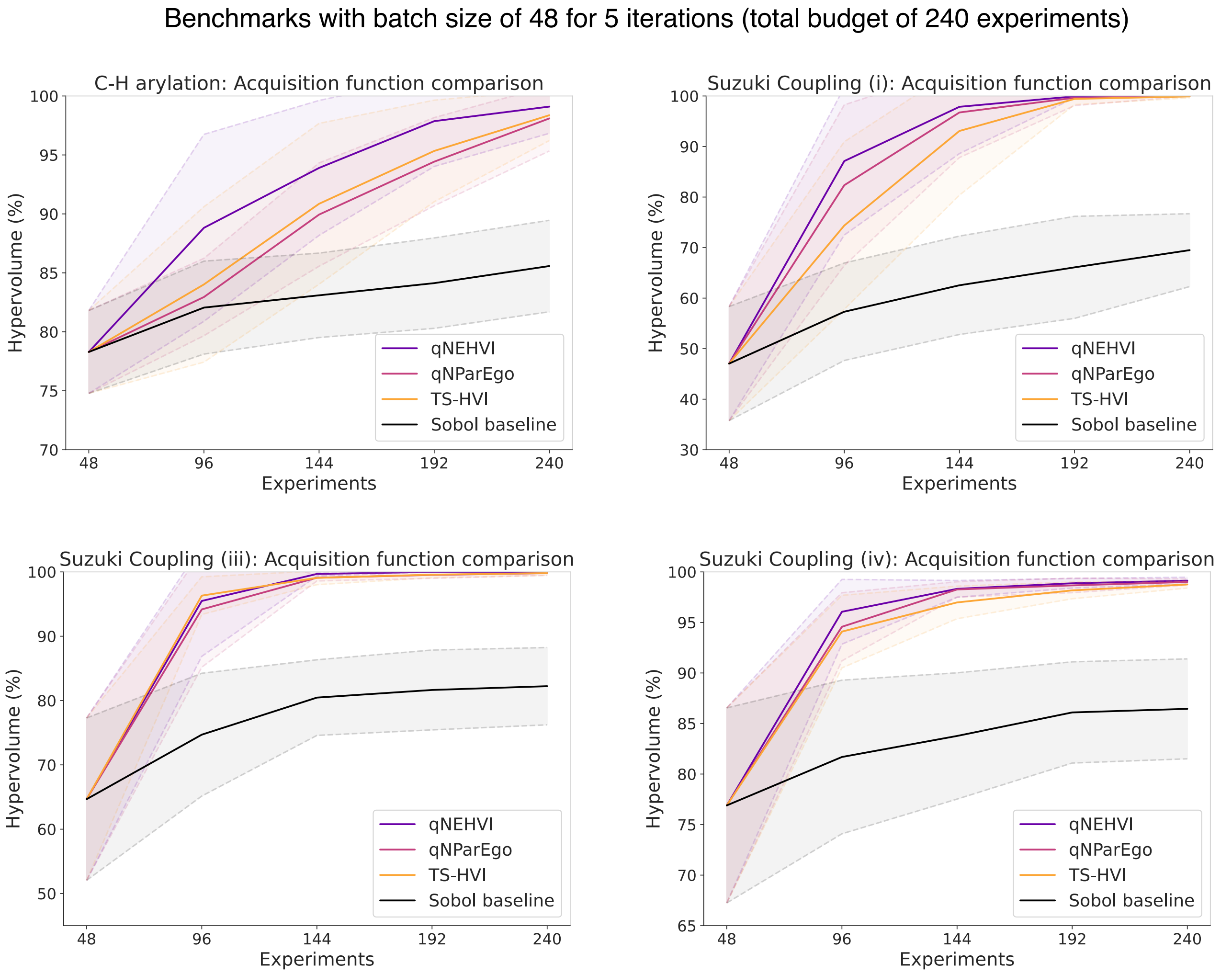

Supplementary Figure 4: Optimisation algorithm performance with batch size 48 on all benchmark datasets. Repeated across 20 different random seeds with plotted hypervolume (%) mean and ± 1 standard deviation.

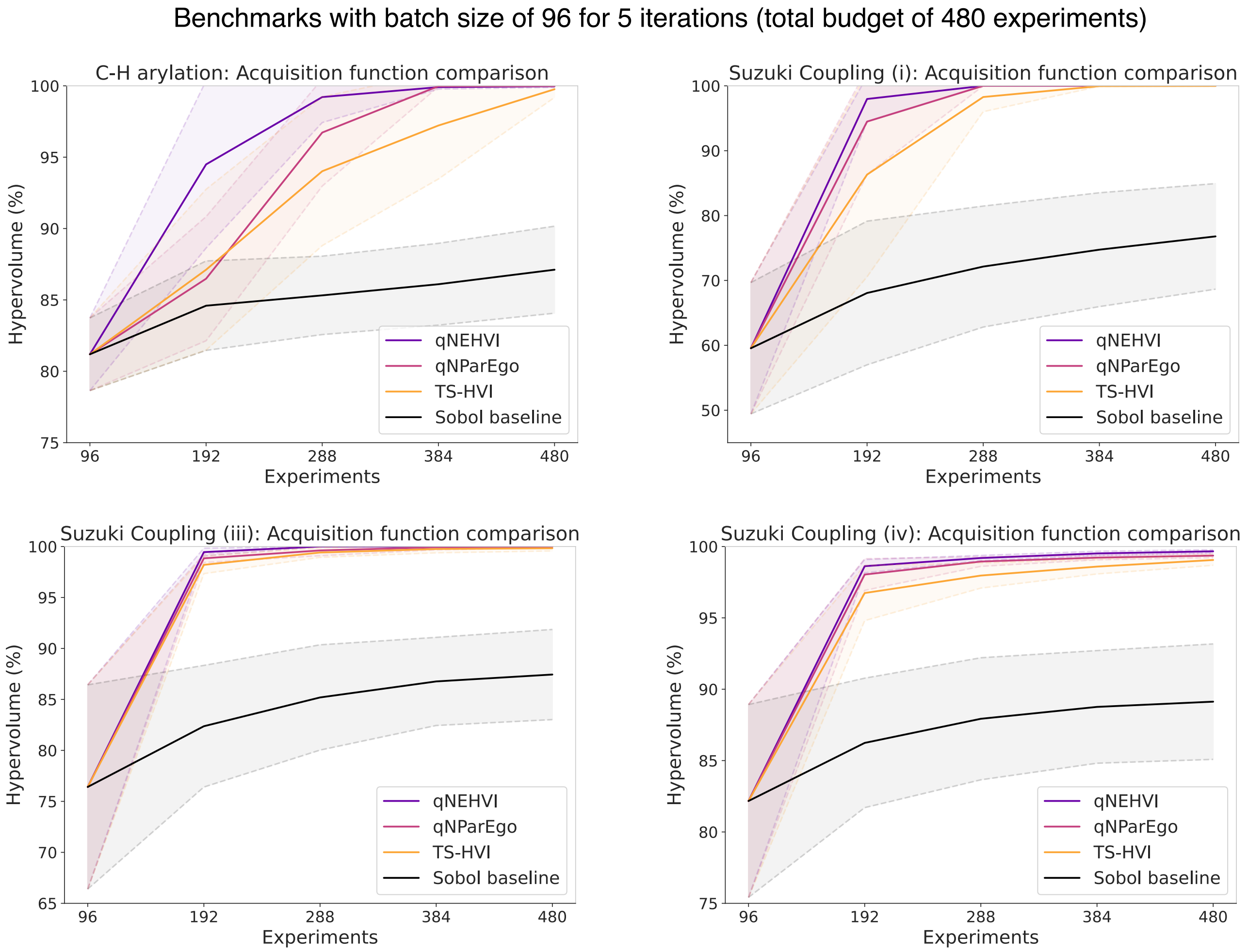

Supplementary Figure 5: Optimisation algorithm performance with batch size 96 on all benchmark datasets. Repeated across 20 different random seeds with plotted hypervolume (%) mean and ± 1 standard deviation.

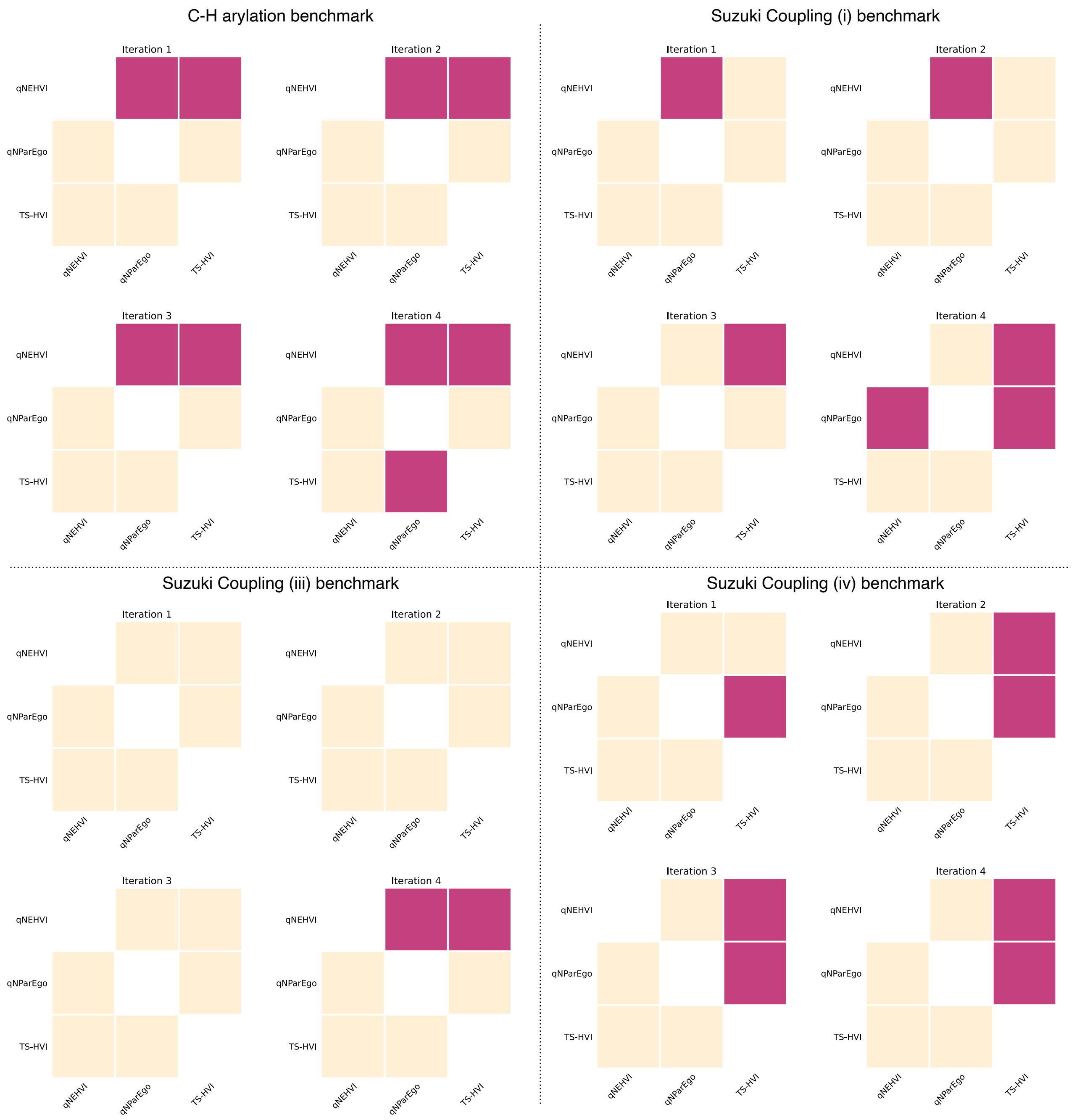

Supplementary Figure 6: Statistical significance plots comparing the different acquisition functions on the benchmark datasets across 20 random seeds. An entry is shaded if the ith row algorithm statistically significantly outperforms the jth column algorithm on a Wilcoxon one-sided paired test [4] implemented using a 5% significance level.

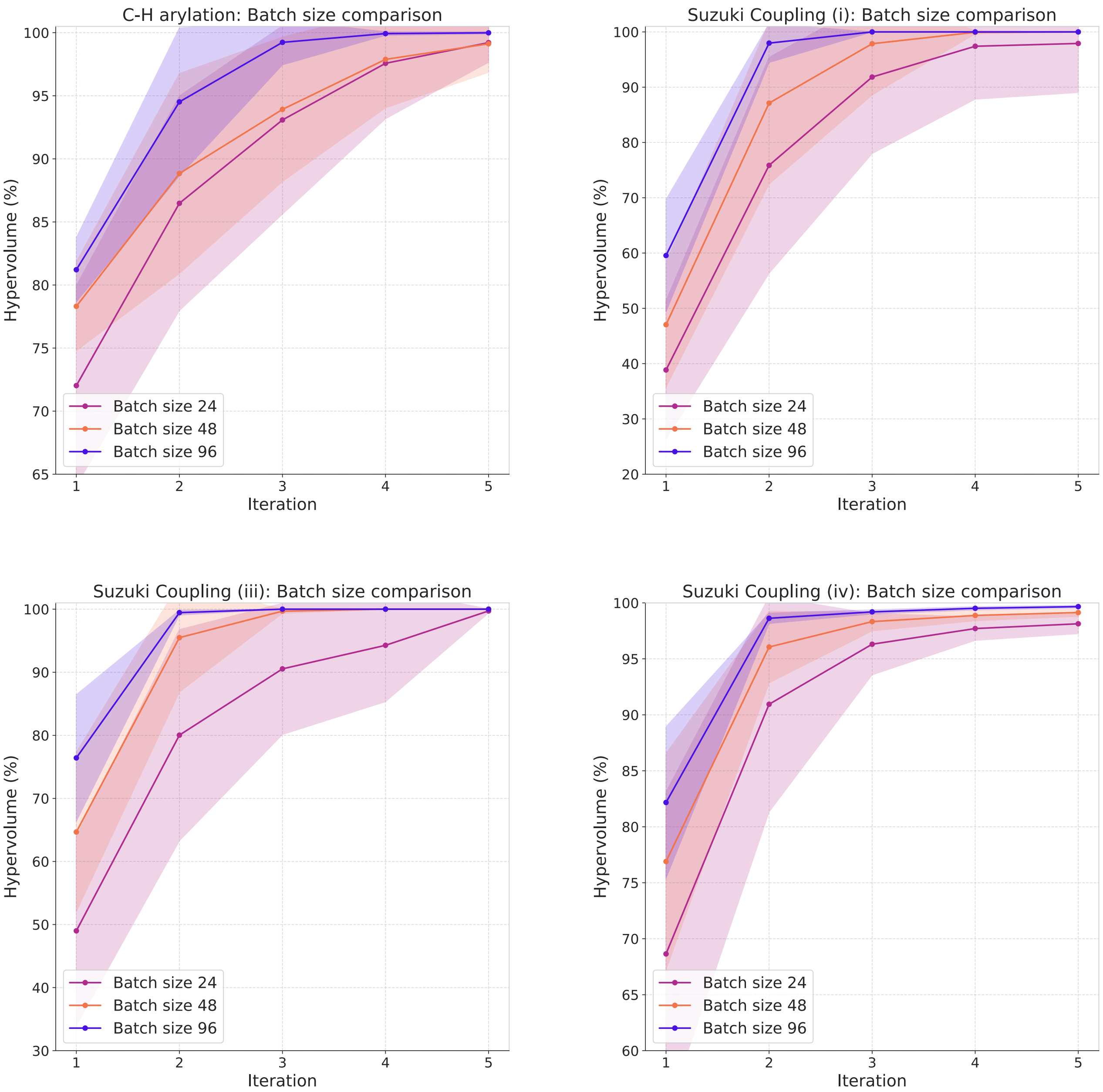

Supplementary Figure 7: Optimisation performance comparison of our workflow (q-NEHVI acquisition function) across different batch sizes (24/48/96) on all benchmark datasets. Each iteration represents one week of HTE experimental cycle time. Repeated across 20 different random seeds with plotted hypervolume (%) mean and ± 1 standard deviation.

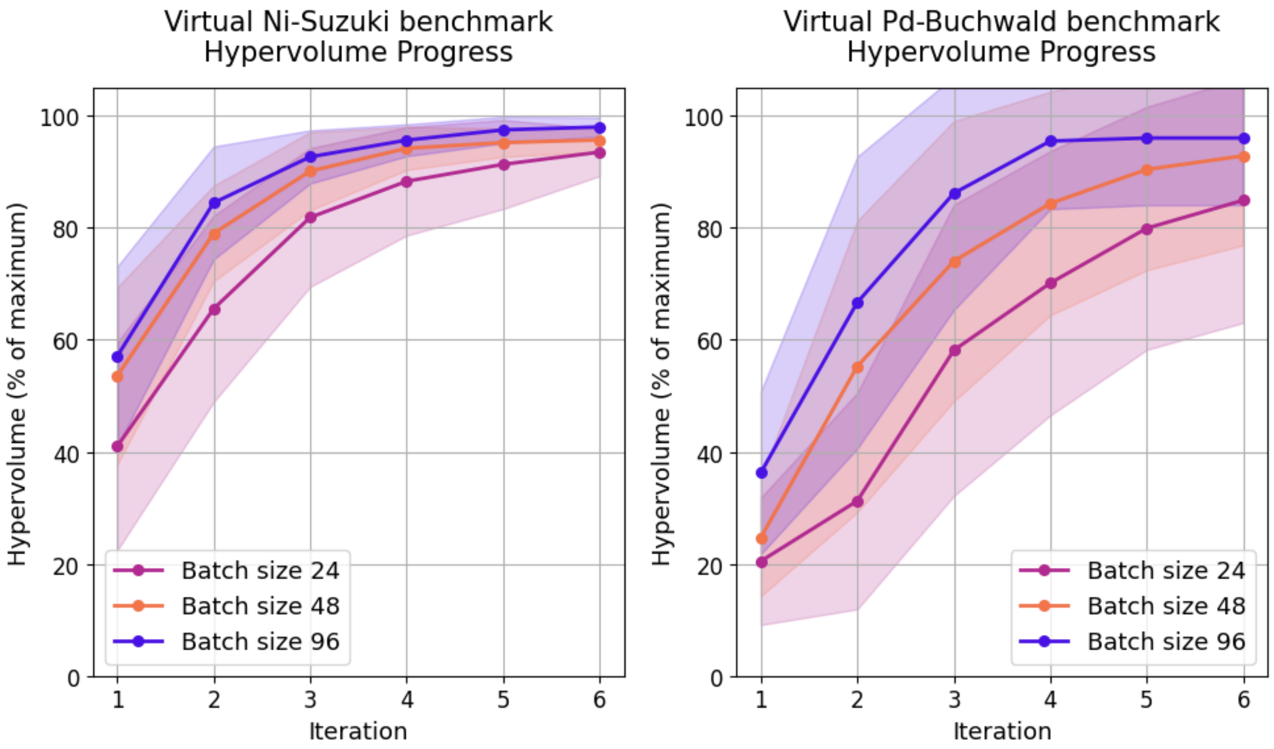

Supplementary Figure 8: Optimisation performance comparison of our workflow (q-NEHVI acquisition function) across different batch sizes (24/48/96) on virtual benchmark datasets generated from our Ni-catalysed Suzuki (88,000 reaction conditions) and API Buchwald-Hartwig (39,600 reaction conditions) experimental case study. Analogous to the other benchmark datasets, we generated these virtual benchmarks using ML regressors trained on experimental data obtained in this work. Each iteration represents one week of HTE experimental cycle time. Repeated across 20 different random seeds with plotted hypervolume (%) mean and ± 1 standard deviation.

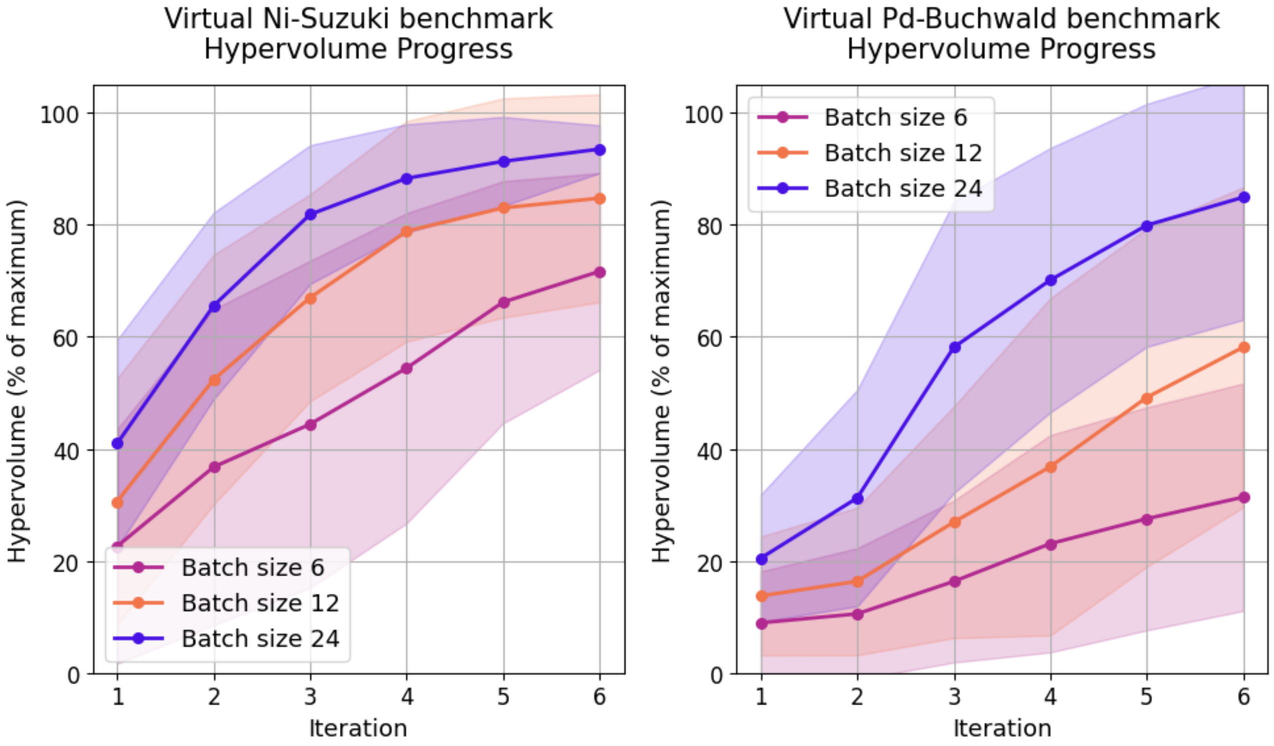

Supplementary Figure 9: Optimisation performance comparison of our workflow (q-NEHVI acquisition function) across different batch sizes (6/12/24) on virtual benchmark datasets generated from our Ni-catalysed Suzuki (88, 000 reaction conditions) and API Buchwald-Hartwig (39, 600 reaction conditions) experimental case study. Analogous to the other benchmark datasets, we generated these virtual benchmarks using ML regressors trained on experimental data obtained in this work. Repeated across 20 different random seeds with plotted hypervolume (%) mean and $\pm$ 1 standard deviation.

As a comparison to our approach, we include here optimisation performance of EDBO+ [1] on the expanded C-H arylation virtual benchmark dataset, which was generated from experimental data provided in EDBO+. To assess computational scalability, several batch sizes were tested on both CPU and GPU for 120 total experiments (see Methods section), the same total number of experiments as our lowest batch setting of 5 iterations with batch size 24. On low batch sizes ≤ 5, we observed that the EDBO+ optimisation was terminated due to memory issues before reaching 120 total experiments when running with both CPU and GPU (Supplementary Figure 10).

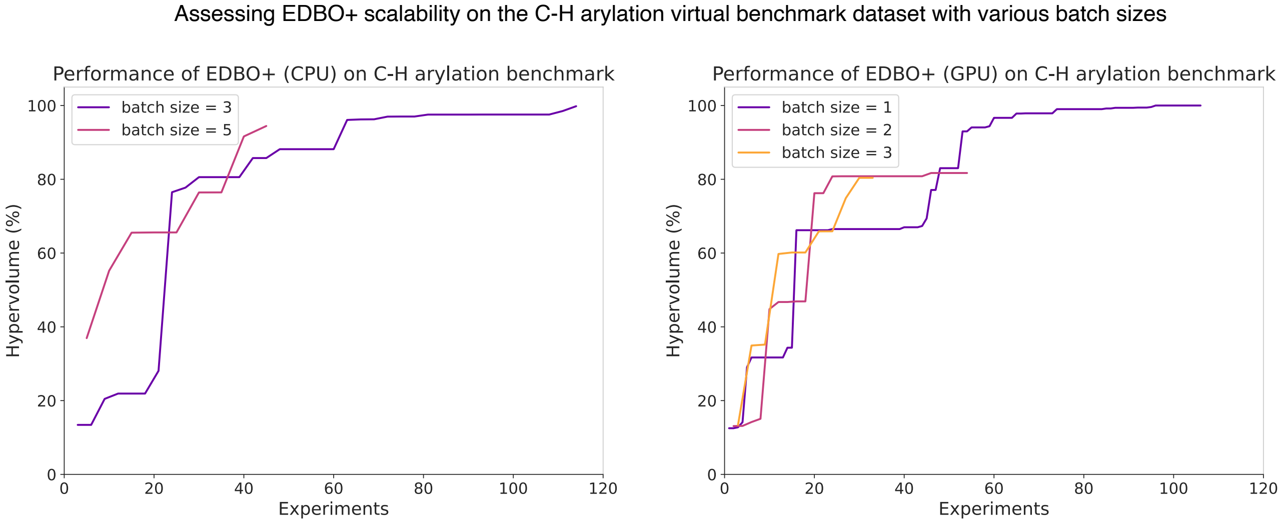

Supplementary Figure 10: Optimisation performance of EDBO+ [1] on the C-H arylation virtual benchmark dataset with various batch sizes for 120 total experiments. Discontinuities where the graph lines stop before 120 total experiments indicate when the EDBO+ optimisation was terminated on our workstation due to memory issues.

3 Noisy benchmarks

We include here all benchmarks with the machine learning (ML) workflow receiving noisy objective values as input (Supplementary Figure 11). These benchmarks intend to demonstrate the ability of our workflow to remain robust under noisy objective (e.g. yield) values, as chemical reactions exhibit some degree of stochasticity in practice. Gaussian noise with varying standard deviations from 2.5 to 10 was added to the objective values, implemented using PyTorch [5]. Noisy benchmarks were run using the qNEHVI acquisition function with 5 iterations of batch size 24. Only yield and turnover values were perturbed across all datasets, neglecting the cost objective in the C-H arylation benchmark dataset. The noisy values were clamped at 0 and 100 for yield (%), and for turnover, at 0 and the max observed values.

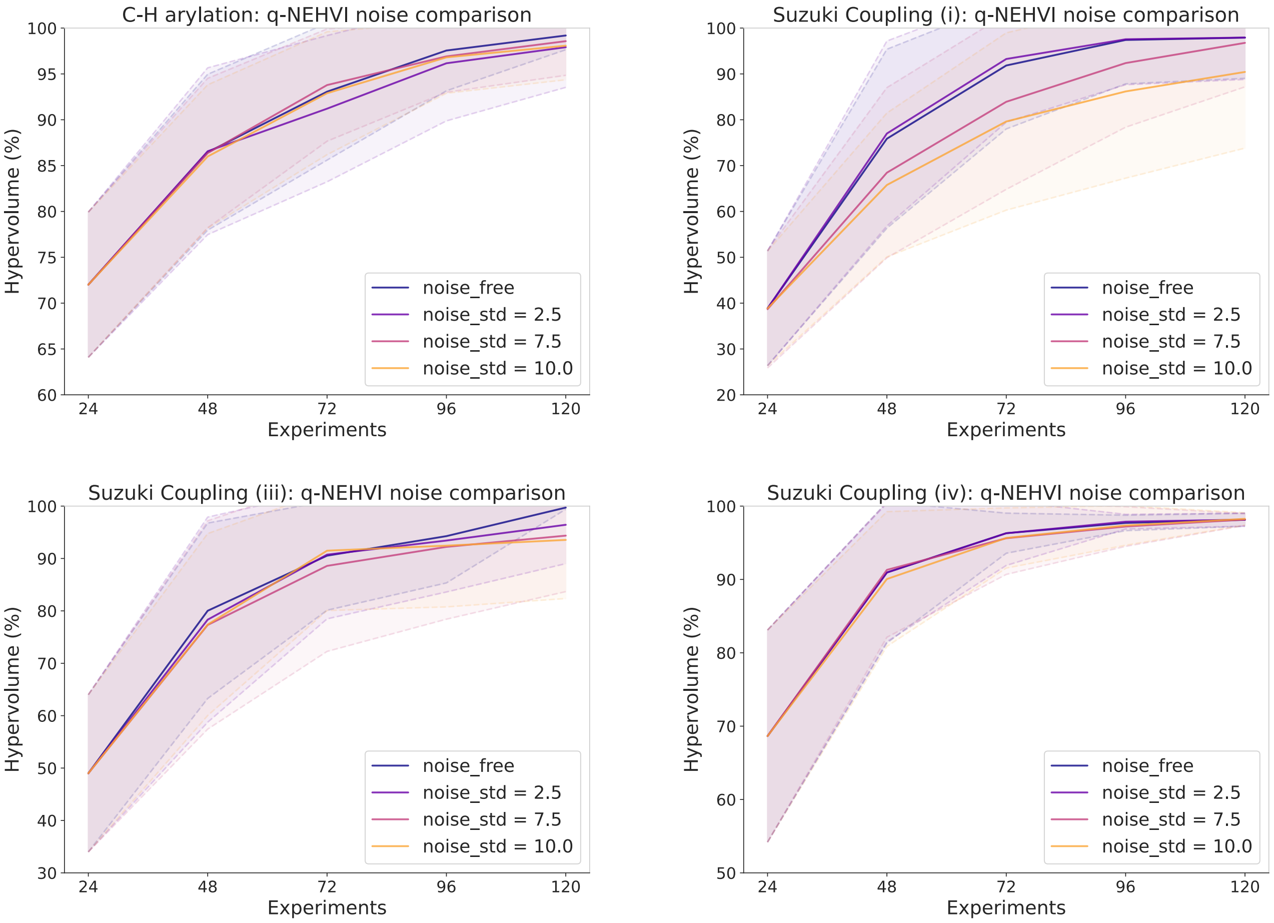

Supplementary Figure 11: Optimisation performance with varying degrees of added input noise on all benchmark datasets with 5 iterations of batch size 24. Repeated across 20 different random seeds with plotted hypervolume (%) mean and $\pm$ 1 standard deviation.

4 Constrained benchmarks

For the naive and nested constrained strategies (see main text Equipment Constraints on Experimental Batches section and Methods for explanation), we detail here optimisation results on all benchmark datasets, showing optimisation results under the restrictions of two and one unique temperature(s) per batch of optimisation (Supplementary Figure 12). Initialisation data in these scenarios were also restricted to two or one unique randomly selected temperatures according to the set constraints of the campaign. Constrained benchmarks were run using the qNEHVI acquisition function with 5 iterations of batch size 24. Supplementary Figures 13 and 14 show statistical significance plots comparing the performance of nested and naive algorithms under batch constraints of 1 and 2 unique temperatures, respectively. We observe that the nested algorithm outperforms the naive algorithm under the lowest batch constraint of 1 unique temperature per batch.

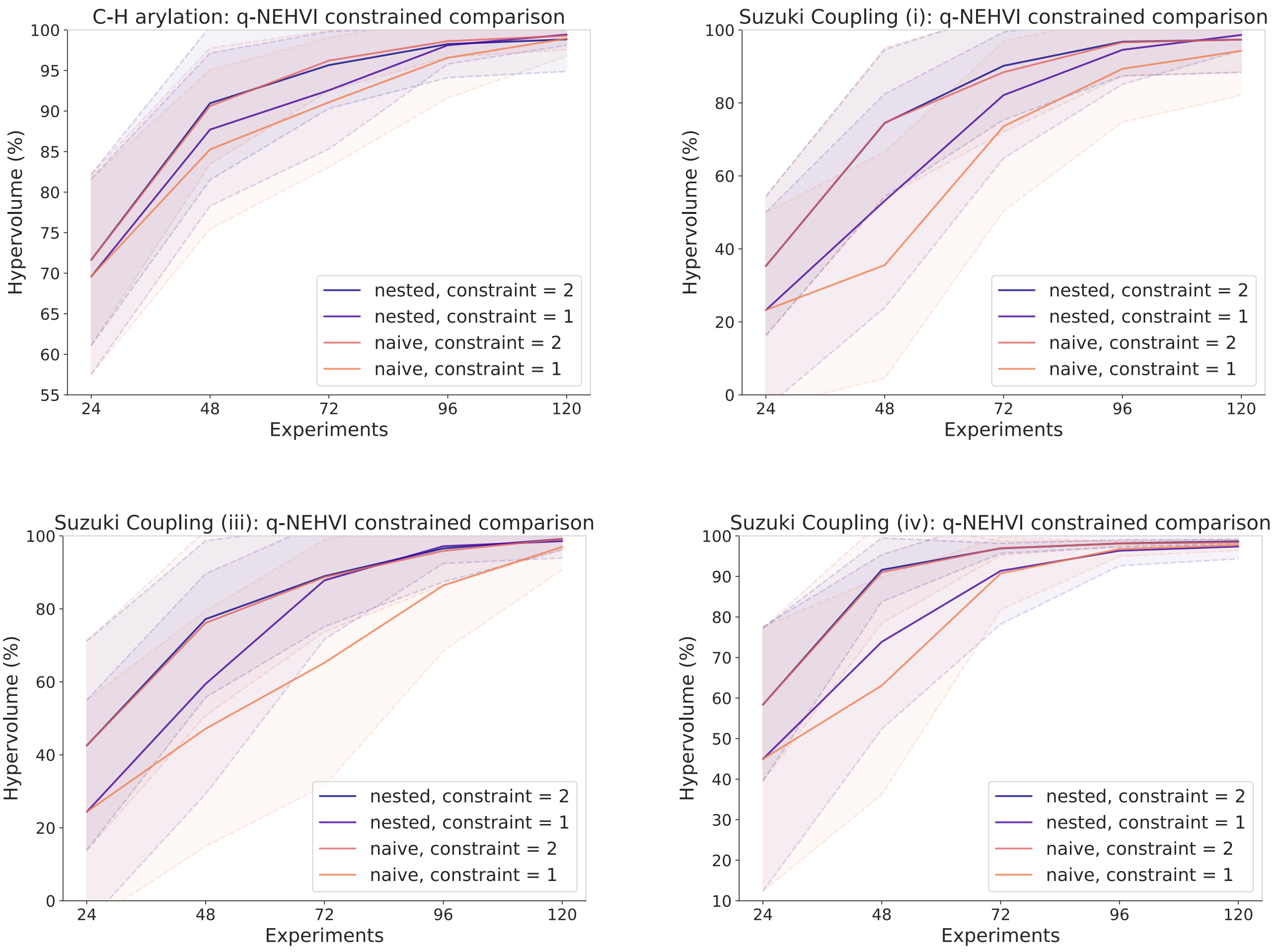

Supplementary Figure 12: Optimisation performance with varying batch constraints on all benchmark datasets with 5 iterations of batch size 24. Repeated across 20 different random seeds with plotted hypervolume (%) mean and ± 1 standard deviation.

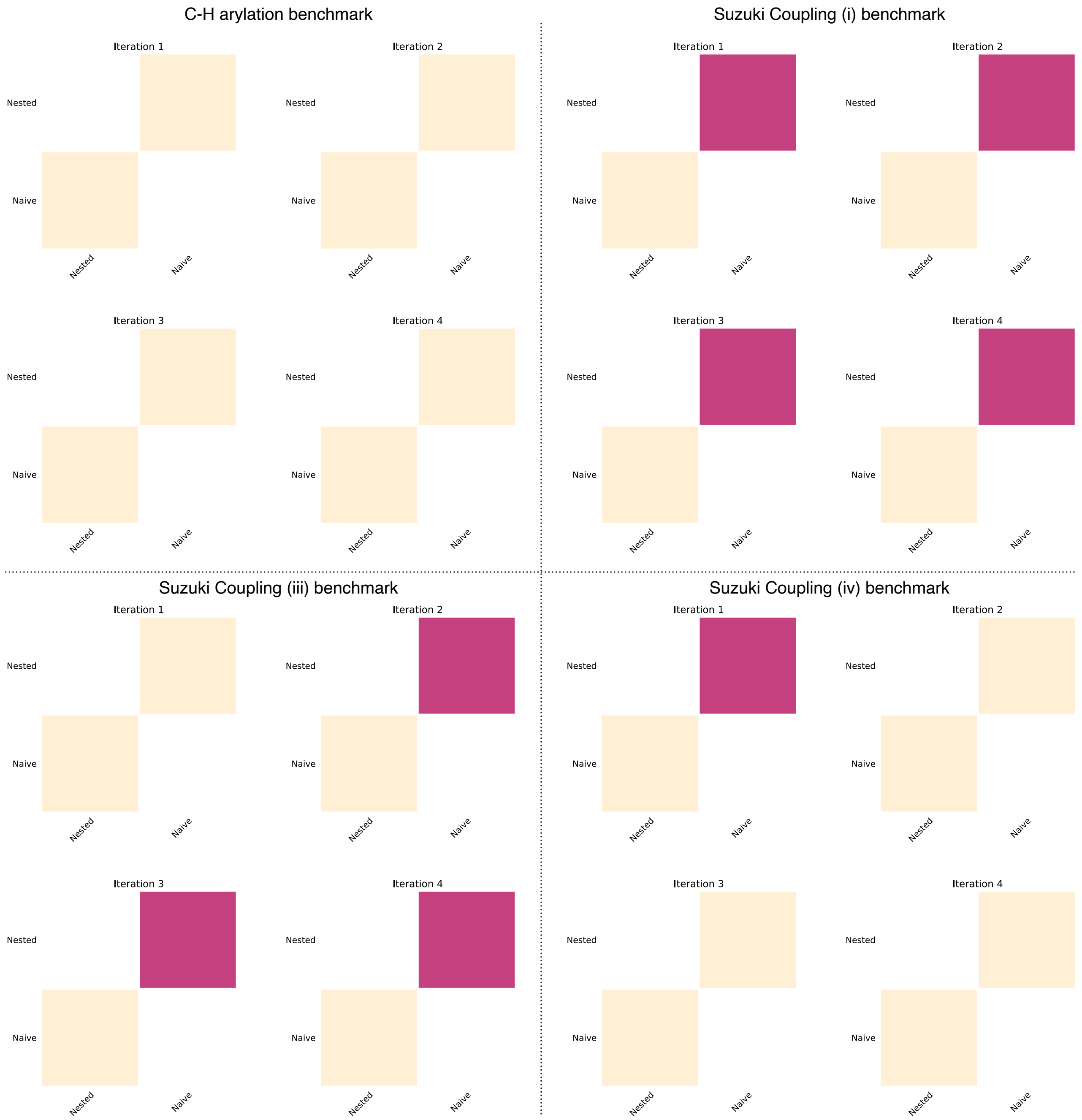

Supplementary Figure 13: Statistical significance plots comparing the constrained strategies on the benchmark datasets with constraint of one temperature across 20 random seeds. An entry is shaded if the ith row algorithm statistically significantly outperforms the jth column algorithm on a Wilcoxon one-sided paired [4] test implemented using a 5% significance level.

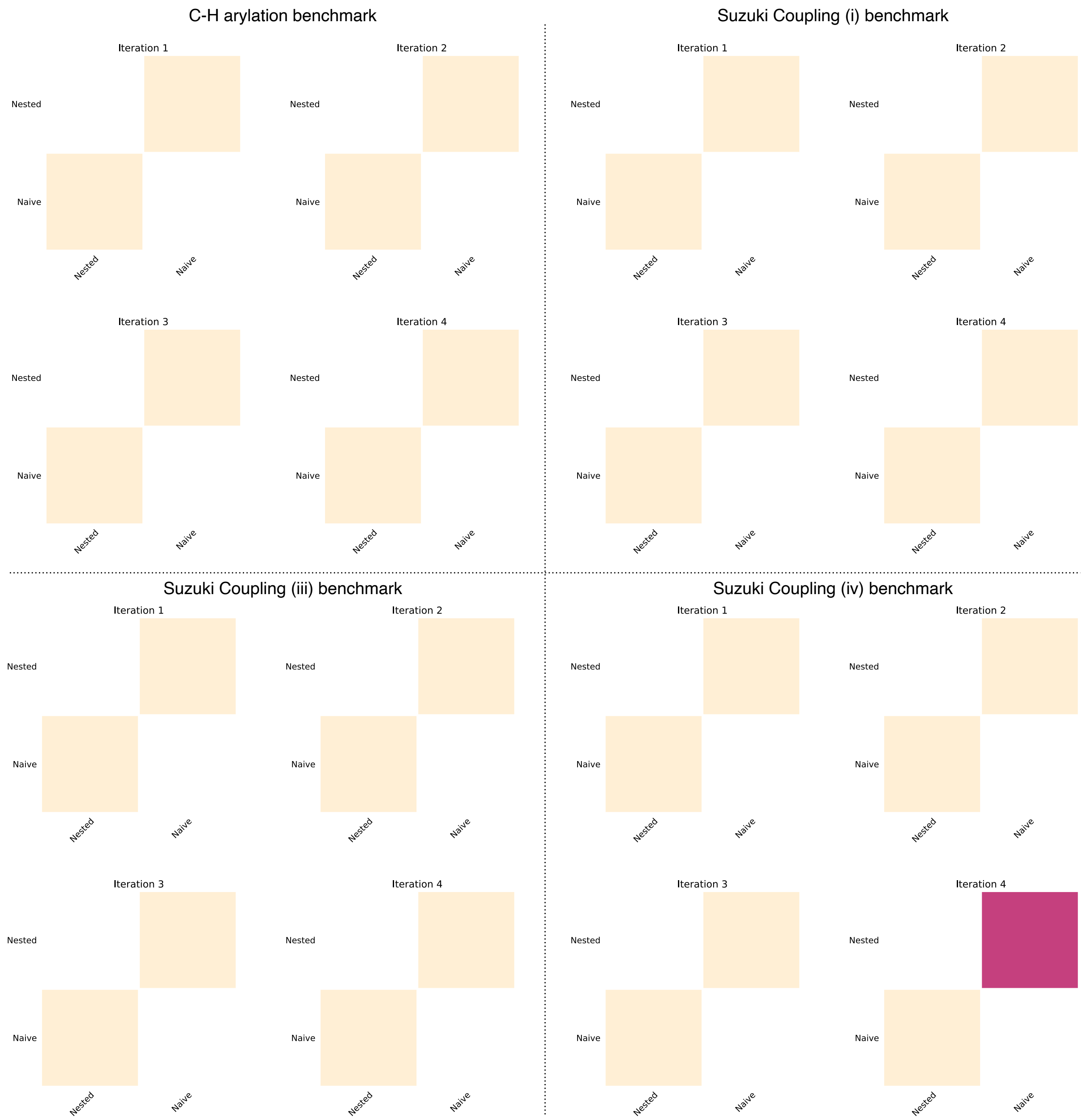

Supplementary Figure 14: Statistical significance plots comparing the constrained strategies on the benchmark datasets with constraint of two temperatures across 20 random seeds. An entry is shaded if the ith row algorithm statistically significantly outperforms the jth column algorithm on a Wilcoxon one-sided paired test [4] using a 5% significance level.

5 Experimental section

5.1 HTE platform

High-throughput experiments were conducted using a parallel experimentation platform custom-designed by UnchainedLabs (Supplementary Figure 15). The system comprises two interconnected Big Kahuna platforms, one of which is further integrated with a LiCONiC LiCotel system. The entire setup is encased within an LC Technology Solutions glove box featuring dual circulation systems—one dedicated to solid dispensing and another to reaction execution. LCMS analysis was performed using an ACQUITY UPLC I-Class system with QDa from Waters.

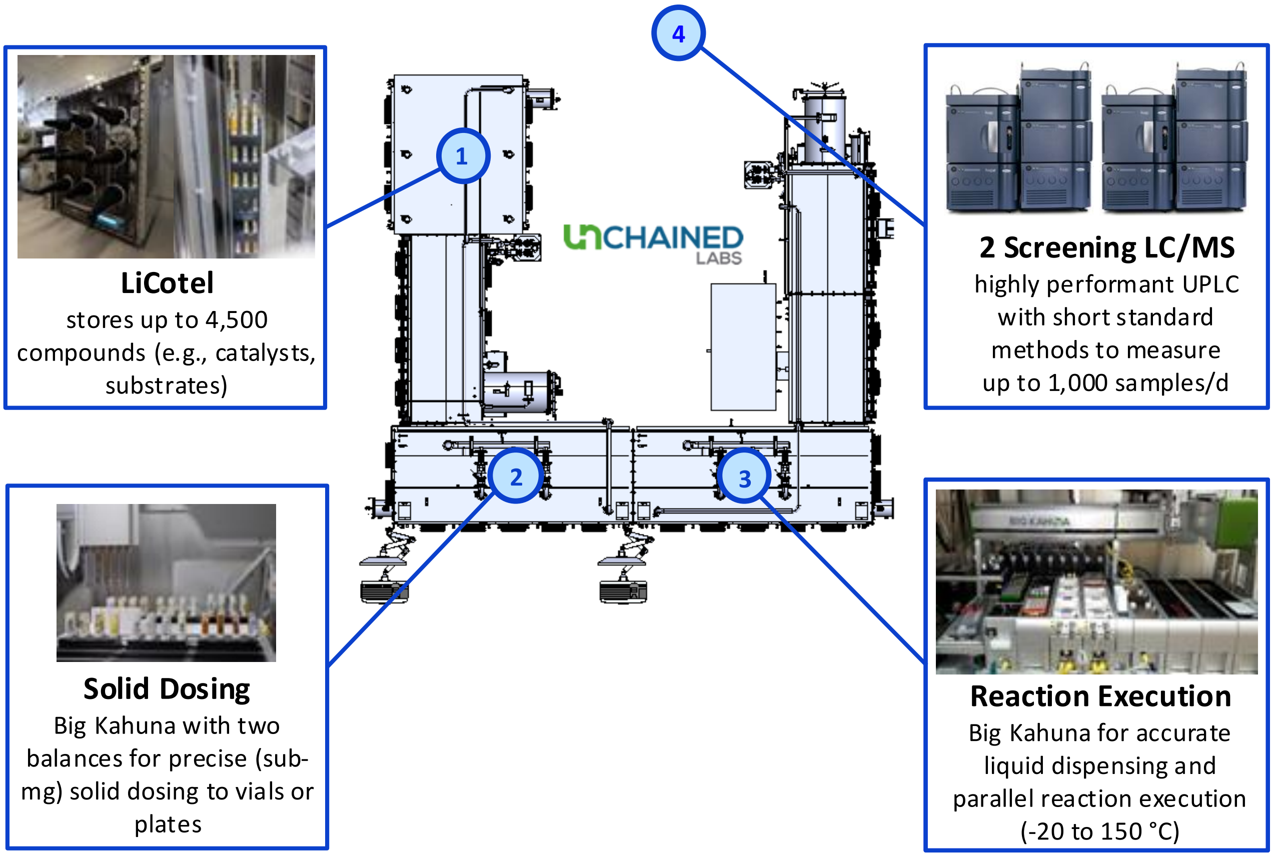

Supplementary Figure 15: Schematic overview of high-throughput experimental set-up.

Solid components were dispensed using a Mettler balance in vial dispense mode, utilising SV hoppers for precursors, ligands, and substrates, while 10 mL classic hoppers were used for solid additives. For target dispense quantities < 0.4 mg, materials were dispensed as coated ChemBeads (e.g., Pd precursors for the API study). Following automated solid dispensing, liquid components (substrates, liquid additives, and solvents) were manually added using an Eppendorf Multipette E3 single channel pipet (4987000010). Reactions were performed in standard 96-position parallel synthesis reaction blocks (Analytical Sales and Services, SKU: 96960), with V&P Scientific super tumble stir discs (VP 721F-1) for stirring.

Data analysis was performed using the HTE OS workflow described by Wuitschik et al. [6] HTE OS is integrated with a SpotFire application that enables tagging of LCMS signals into categories (e.g., "limiting SM", "other SM", "solvent", "ignore peak"). During analysis, all peaks were tagged accordingly, with peaks corresponding to ligands and precatalyst components designated as "ignore peak".

For LCAP calculations, signals tagged as “other SM”, “solvent”, or “ignore peak” were excluded from consideration.

5.2 Nickel-catalysed Suzuki-Miyaura coupling: 7-Methyl-2-(5-pyrimidyl)pyrrolo[2,3-d]pyrimidine

5.2.1 Reaction condition search space

We include a full list of reaction parameters in the reaction condition search space for the Ni-catalysed Suzuki reaction experimental study detailed in the main text (Supplementary Figure 16). The combinatorial set of reaction parameter combinations encompassed 120,000 possible configurations, and after removing configurations for which the reaction temperature exceeded the solvent boiling point, we obtained 88,000 possible reaction conditions.

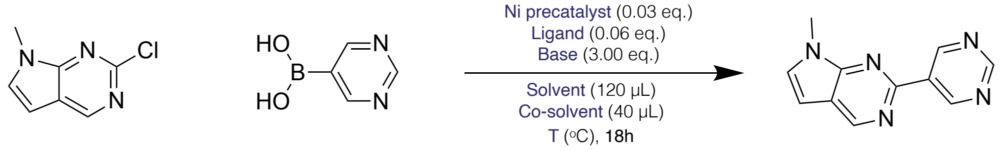

1 (10.0 mg) — **2** (9.6 mg, 1.3 eq.) — 88,000 total practical combinations — **3**

Monophosphine ligand (50)

PPh_3	cataCXium A	Me_4tBuXPhos	$CyPPh_2$	GPhos
PCy_3	cataCXium POMetB	JackiePhos	DavePhos	Tyrannophos
$P(oTol)_3$	cataCXium POMeCy	BrettPhos	EPhos	CM-Phos
$P(2\text{-Fur})_3$	vBRIDP	SPhos	PhenCar-Phos	PCy_2Ph
JohnPhos	Cy-vBRIDP	PhDavePhos	CPhos	$NPCy_2$-Phendolephos
$P(4\text{-OMe-}C_6H_4)_3$	tBuDavePhos	CyAmPhos	PhCPhos	Amidolephos
$P(2,4,6\text{-TriMeO})_3$	CyJohnPhos	AmPhos	(tBu)PhCPhos	$PtBuPh_2$
$P(4\text{-CF}_3\text{-}C_6H_4)_3$	cataCXium PICy	BippyPhos	PAd_3	$PtBuCy_2$
$P(2\text{-OMe-}C_6H_4)_3$	XPhos	CyBippyPhos	cataCXium PiPr	iPrPhenCar-Phos
cataCXium ABn	MorDalphos	RuPhos	EtPhenCar-Phos	PhXPhos

Solvents (10)

MeTHF, MeCN, PhMe, iPrOh, DEC, EtOAc, MEK, MeOH, tAmOH, DMI

**Nickel precatalyst** (4)	**Base** (4)	**Co-solvent** (3)	**Temperature** (°C) (5)
[Ni(oTol)(Cl)(TMEDA)]	DIPEA	None	40
$[Ni(oTol)(Cl)(PPh_3)_2]$	DBU	H_2O	55
[Ni(COD)(DQ)]	Cs_2CO_3	MeOH	70
$NiCl_2.6H_2O$	K_3PO_4		85
			100

Supplementary Figure 16: Full reaction condition space for the Ni-catalysed Suzuki reaction experimental case study using our ML optimisation workflow.

5.2.2 Experimental procedure for HTE campaign with ML optimisation workflow

Solid ligands (3.58 µmol, 6.0 mol%), nickel precursor (1.79 µmol, 3.0 mol%), solid bases (179 µmol, 3.0 eq), 2-chloro-7-methyl-pyrrolo[2,3-d]pyrimidine (10.0 mg, 59.7 µmol) and 4-pyrimidylboronic acid (9.6 mg, 77.5 µmol, 1.3 eq) were dispensed into 1 mL vials with stirring disks in a 96 well plate. Liquid ligands (3.58 µmol, 6.0 mol%), solvents (120 µL), co-solvents (40 µL), and liquid bases (179 µmol, 3.0 eq) were added and the vials separated into two plates based on the reaction temperature. The plates were sealed and stirred at the indicated temperature for 20 h. The plates were then recombined, 4/1

MeCN/H_2O (500 µL) was added and shaken for 20 min at rt. 25 µL samples taken and analysed by Liquid Chromatography-Mass Spectroscopy (LC-MS) to obtain area percent (AP) metrics.

5.2.3 Experimental procedure for HTE campaign with experimentalist-designed HTE plates

For the first experimentalist-designed HTE plate (Supplementary Figure 17), ligands (9.85 µmol, 11.0 mol%), [Ni(COD)(DQ)] (1.23 mg, 4.48 µmol, 5.0 mol%), K_2CO_3 (37.1 mg, 268 µmol, 3.0 eq), 2-chloro-7-methyl-pyrrolo[2,3-d]pyrimidine (15.0 mg, 89.5 µmol) and 4-pyrimidylboronic acid (16.7 mg, 134 µmol, 1.5 eq) were dispensed into 1 mL vials with stirring disks in a 96 well plate. Solvents (225 µL), water (45 µL), and DIPEA (44.3 µL, 268 µmol, 3.0 eq) were added. The plate was sealed and stirred at 70 °C for 18 h. MeOH (200 µL) added and shaken vigorously for 10 min at rt. 25 µL samples taken and analysed by Liquid Chromatography-Mass Spectroscopy (LC-MS) to obtain area percent (AP) metrics.

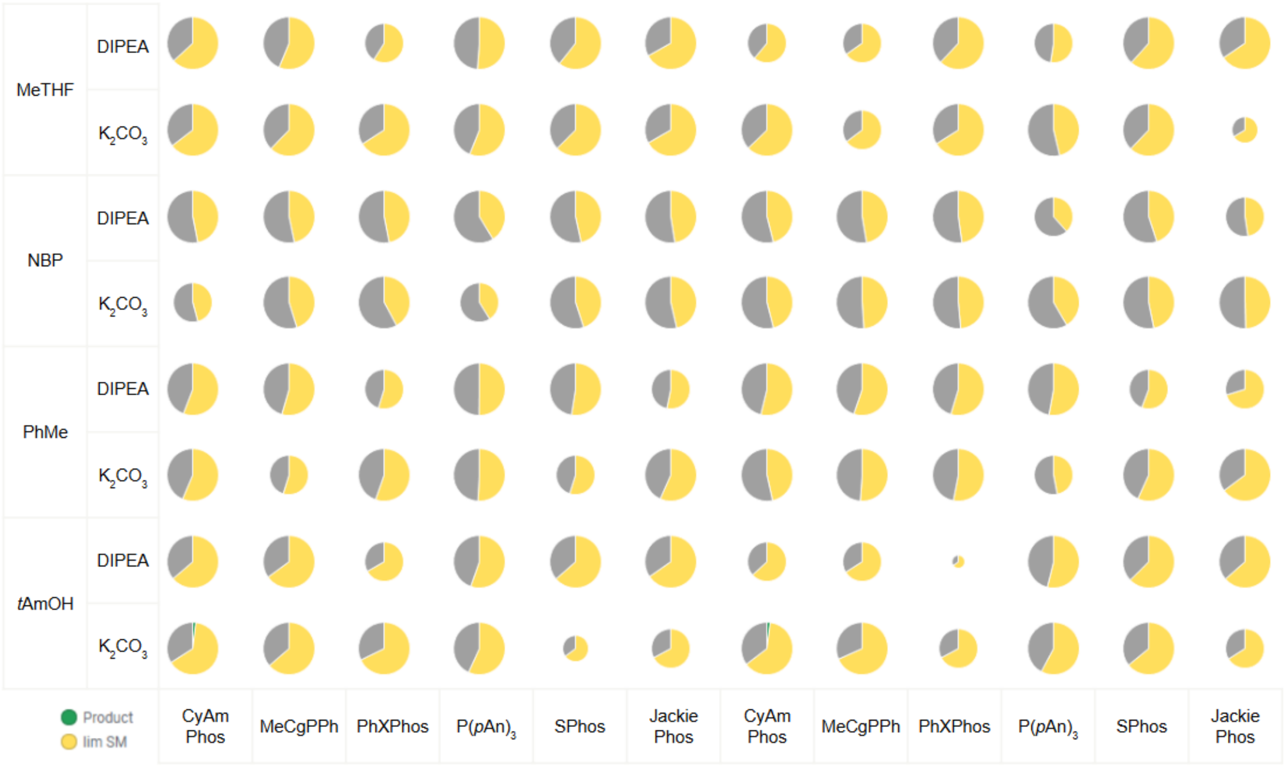

Supplementary Figure 17: Plate layout of first chemist-designed plate (Ni-catalysis), visualised by the HTE OS workflow [6]. Pie charts show area percent of substances in each well.

For the second experimentalist-designed HTE plate (Supplementary Figure 18), ligands (8.75 µmol, 11.0 mol%), [NiCl(oTol)(TMEDA)] (1.20 mg, 3.97 µmol, 5.0 mol%), K_2CO_3 (33.0 mg, 238 µmol, 3.0 eq), 2-chloro-7-methyl-pyrrolo[2,3-d]pyrimidine (13.3 mg, 79.5 µmol) and 4-pyrimidylboronic (14.8 mg, 119 µmol, 1.5 eq) were dispensed into 1 mL vials with stirring disks in a 96 well plate. Solvents (200 µL), water (40 µL), and DIPEA (39.4 µL, 238 µmol, 3.0 eq) were added. The plate was sealed and stirred at 85 °C for 20 h. MeOH (200 µL) added and shaken vigorously for 10 min at rt. 25 µL samples taken and analysed by Liquid Chromatography-Mass Spectroscopy (LC-MS) to obtain area percent (AP) metrics.

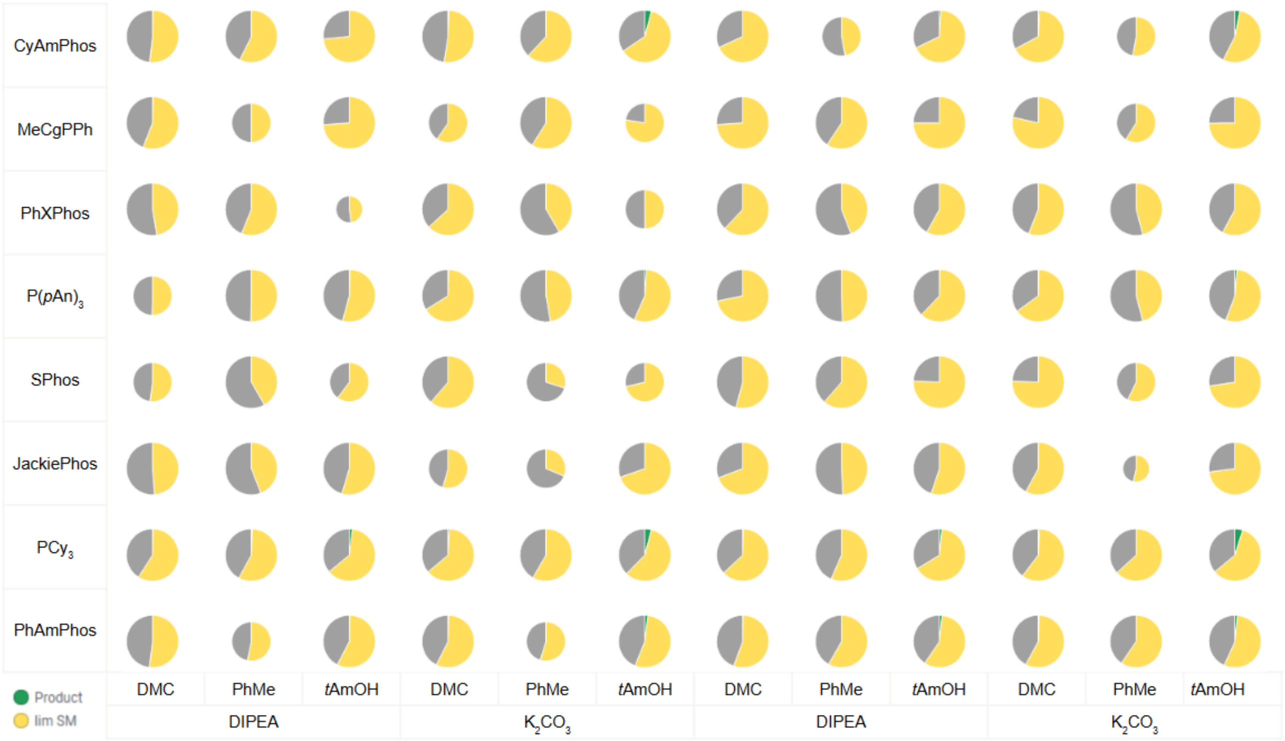

Supplementary Figure 18: Plate layout of second chemist-designed plate (Ni-catalysis), visualised by the HTE OS workflow [6]. Pie charts show area percent of substances in each well.

For the third experimentalist-designed HTE plate (Pd-catalysis) (Supplementary Figure 19), ligands (8.75 µmol, 11.0 mol%), palladium precursor (1.99 µmol, 2.5 mol%), K_2CO_3 (33.0 mg, 238 µmol, 3.0 eq), 2-chloro-7-methyl-pyrrolo[2,3-d]pyrimidine (13.3 mg, 79.5 µmol) and 4-pyrimidylboronic acid (14.8 mg, 119 µmol, 1.5 eq) were dispensed into 1 mL vials with stirring disks in a 96 well plate. Solvents (200 µL), and water (40 µL) were added. The plate was sealed and stirred at 70 °C for 20 h. MeOH (200 µL) added and shaken vigorously for 10 min at rt. 25 µL samples taken and analysed by Liquid Chromatography-Mass Spectroscopy (LC-MS) to obtain area percent (AP) metrics.

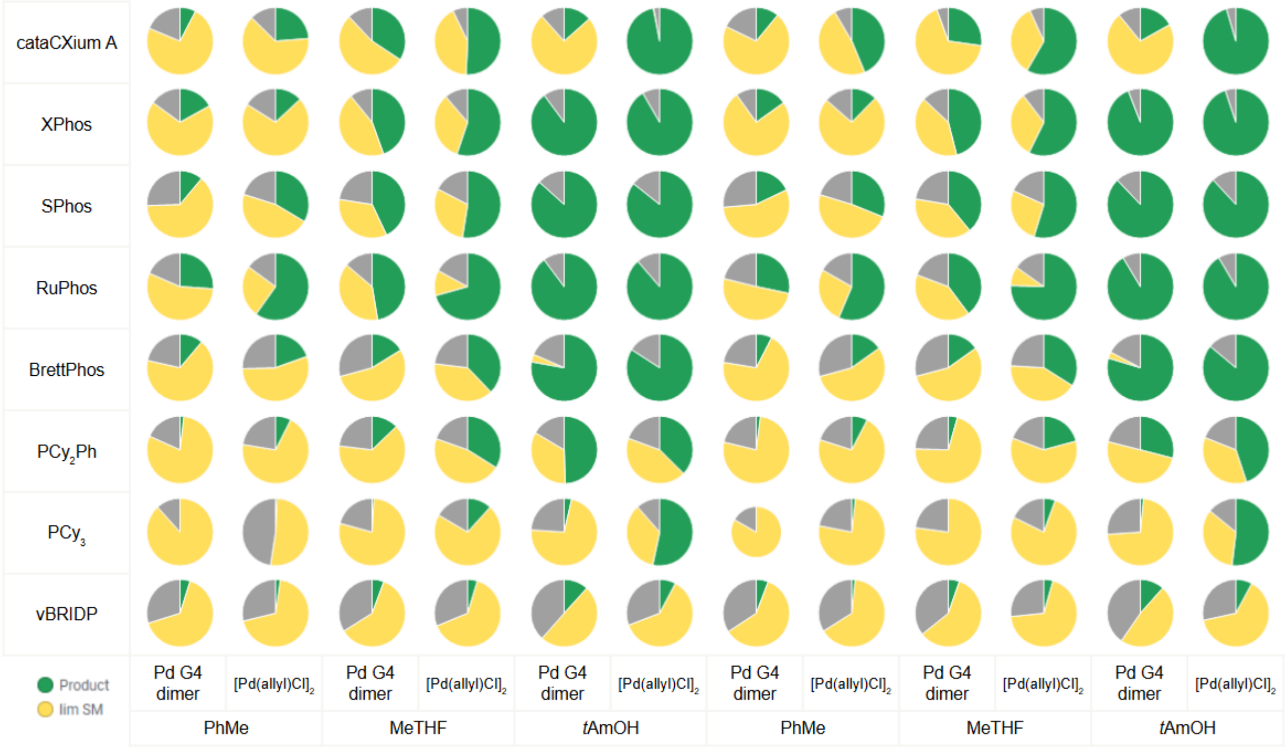

Supplementary Figure 19: Plate layout of third chemist-designed plate (Pd-catalysis), visualised by the HTE OS workflow [6]. Pie charts show area percent of substances in each well.

5.2.4 Scale-up of HTE results: Nickel catalysis

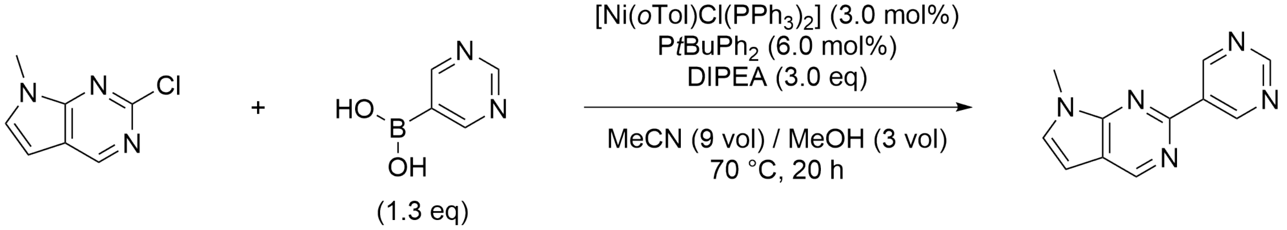

MeOH (5.0 mL) and DIPEA (5.2 mL, 30 mmol, 1.3 eq) were added to 2-chloro-7-methyl-pyrrolo[2,3-d]pyrimidine (1.68 g, 10.0 mmol) and 4-pyrimidylboronic acid (1.61 g, 13.0 mmol, 1.3 eq) in MeCN (10.1 mL) and heated to 70 °C. [Ni(oTol)Cl(PPh_3)$_2$] (213 mg, 300 µmol, 3.0 mol%) and $PtBuPh_2$ (145.4 mg, 600 µmol, 6.0 mol%) were stirred in MeCN (5 mL) for 30 min and the suspension was then added to the reaction mixture and stirred at 70 °C overnight. The reaction was cooled to rt and the solvent was removed under reduced pressure. Sat. aq. $KHCO_3$ solution (50 mL) was added and the mixture was extracted two times with EtOAc (2 × 50 mL) and two times with CH_2Cl_2 (2 × 50 mL). The combined organic phases were dried over Na_2SO_4, filtered and the solvent was removed under reduced pressure. Purification by flash column chromatography on silica gel (CH_2Cl_2:MeOH = 100:0 to 90:10) afforded the product as an off-white solid. Yield: 1.39 g (66%).

^{1}H NMR (400 MHz, $CDCl_3$): δ 9.79 (*s*, 2H, Ar*H*), 9.28 (*s*, 1H, Ar*H*), 9.04 (*s*, 1H, Ar*H*), 7.26 (*d*, $^3J_{H,H'}$ = 3.5 Hz, 1H, Ar*H*), 6.61 (*d*, $^3J_{H,H'}$ = 3.5 Hz, 1H, Ar*H*), 3.95 (*s*, 3H, NC*H$_3$*). (Supplementary Figure 20)

^{13}C{^{1}H} NMR (101 MHz, $CDCl_3$): δ 158.8 (arom.) 156.3 (2C, arom.), 153.3 (arom.), 151.2 (arom.), 149.6 (arom.), 132.0 (arom.), 131.0 (arom.), 118.1 (arom.), 99.9 (arom.), 30.9 (NCH$_3$). (Supplementary Figure 21)

MS (ES$^+$): Calcd. for $C_{11}H_{10}N_5$ m/z = 212.1, found 212.2 $[M+H]^+$.

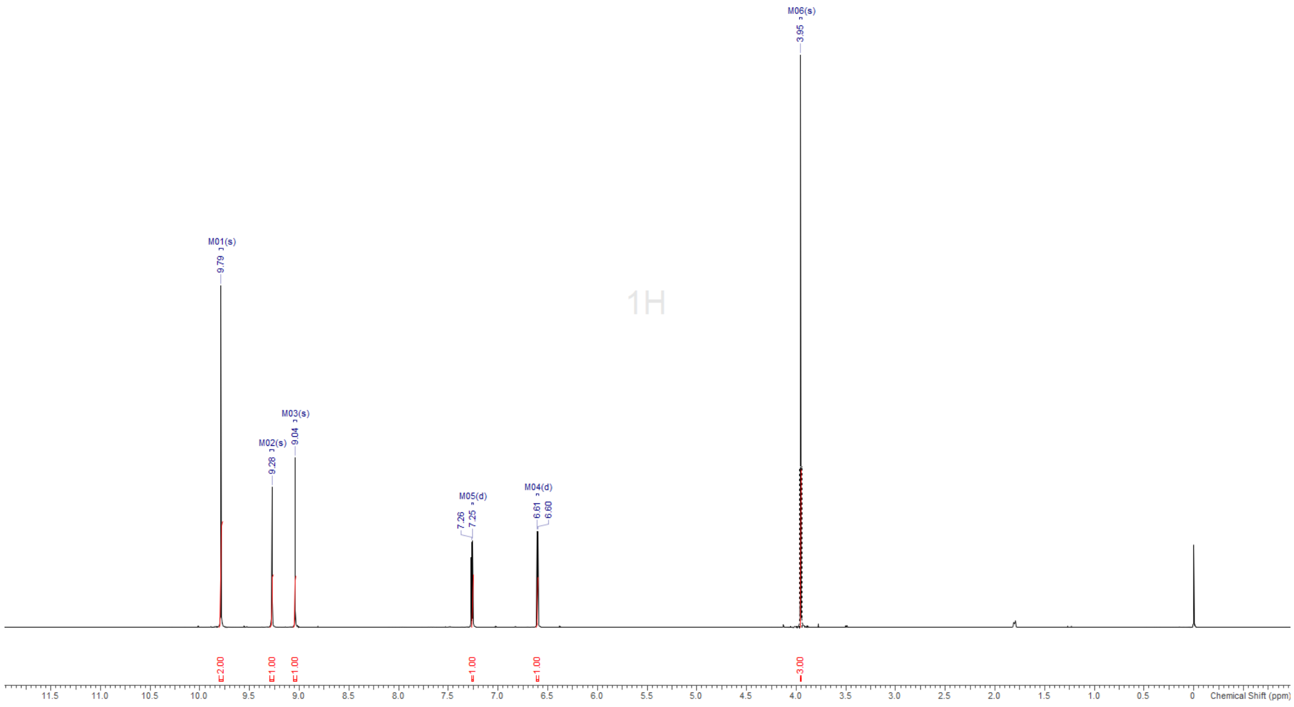

Supplementary Figure 20: Ni-catalysis: ^{1}H NMR (400 MHz, $CDCl_3$) of 7-methyl-2-(5-pyrimidyl)pyrrolo[2,3-d]pyrimidine

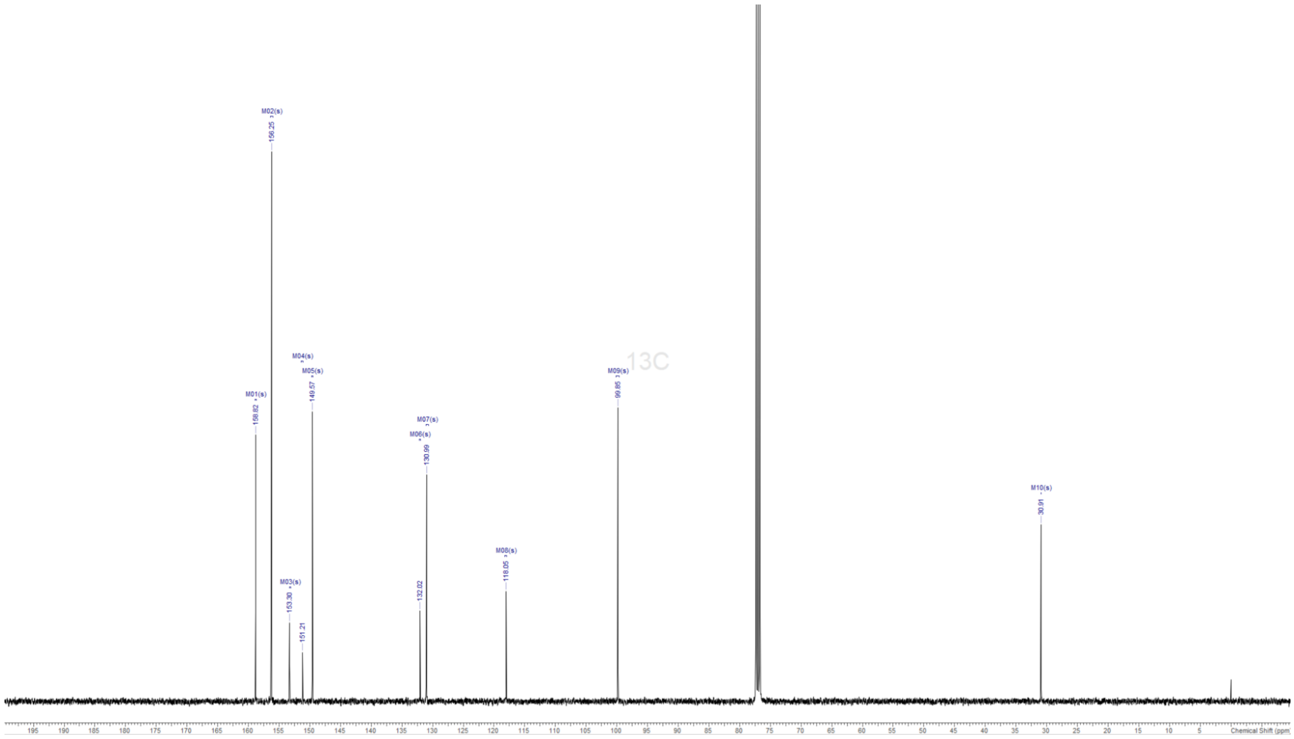

Supplementary Figure 21: Ni-catalysis: $^{13}C\{^1H\}$ NMR (101 MHz, $CDCl_3$) of 7-methyl-2-(5-pyrimidyl)pyrrolo[2,3-d]pyrimidine

5.2.5 Scale-up of HTE results: Palladium catalysis

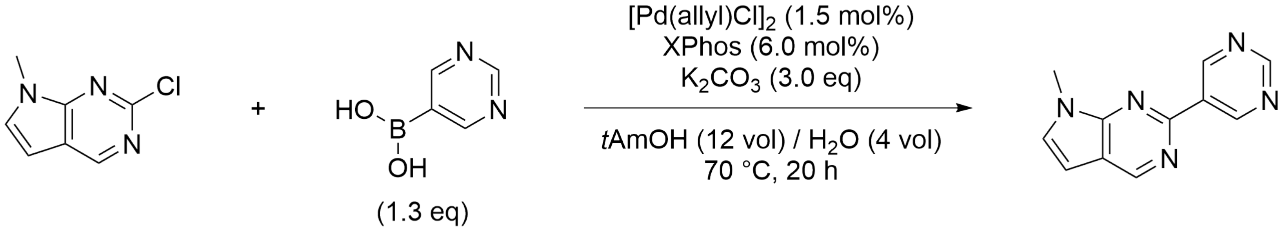

tAmOH (6.00 mL) and H_2O (2 mL) were added to 2-chloro-7-methyl-pyrrolo[2,3-d]pyrimidine (500 mg, 2.98 mmol), 4-pyrimidylboronic acid (481 mg, 3.88 mmol, 1.3 eq), K_2CO_3 (1.24 g, 8.95 mmol, 3.0 eq), $[Pd(allyl)Cl]_2$ (16.4 mg, 44.8 µmol, 1.5 mol%) and XPhos (85.3 mg, 179 µmol, 6.0 mol%) and stirred overnight at 70 °C. The reaction was diluted with EtOAc (20 mL) and water (20 mL) and filtered through Celite. The aqueous phase was separated and extracted twice with EtOAc (2 × 20 mL). The combined organic phases were washed with sat. aq. NaCl solution (20 mL), dried over Na_2SO_4 and the solvent was removed under reduced pressure. Purification by flash column chromatography on silica gel (CH_2Cl_2:MeOH = 100:0 to 90:10) afforded the product as an off-white solid. Yield: 462 mg (73%).

Analytical data in agreement with above data for 7-methyl-2-(5-pyrimidyl)pyrrolo[2,3-d]pyrimidine for nickel catalysis scale-up (Section 5.2.4).

^{1}H NMR (400 MHz, $CDCl_3$): δ 9.79 (*s*, 2H, Ar*H*), 9.27 (*s*, 1H, Ar*H*), 9.04 (*s*, 1H, Ar*H*), 7.26 (*d*, $^3J_{H,H'}$ = 3.5 Hz, 1H, Ar*H*), 6.61 (*d*, $^3J_{H,H'}$ = 3.5 Hz, 1H, Ar*H*), 3.95 (*s*, 3H, NC*H₃*). (Supplementary

Figure 22)

$^{13}C\{^{1}H\}$ NMR (101 MHz, $CDCl_3$): δ 158.8 (arom.) 156.2 (2C, arom.), 153.2 (arom.), 151.2 (arom.), 149.6 (arom.), 132.0 (arom.), 131.0 (arom.), 118.1 (arom.), 99.9 (arom.), 30.9 (NCH_3). (Supplementary Figure 23)

MS (ES^+): Calcd. for $C_{11}H_{10}N_5$ m/z = 212.1, found 212.2 $[M+H]^+$.

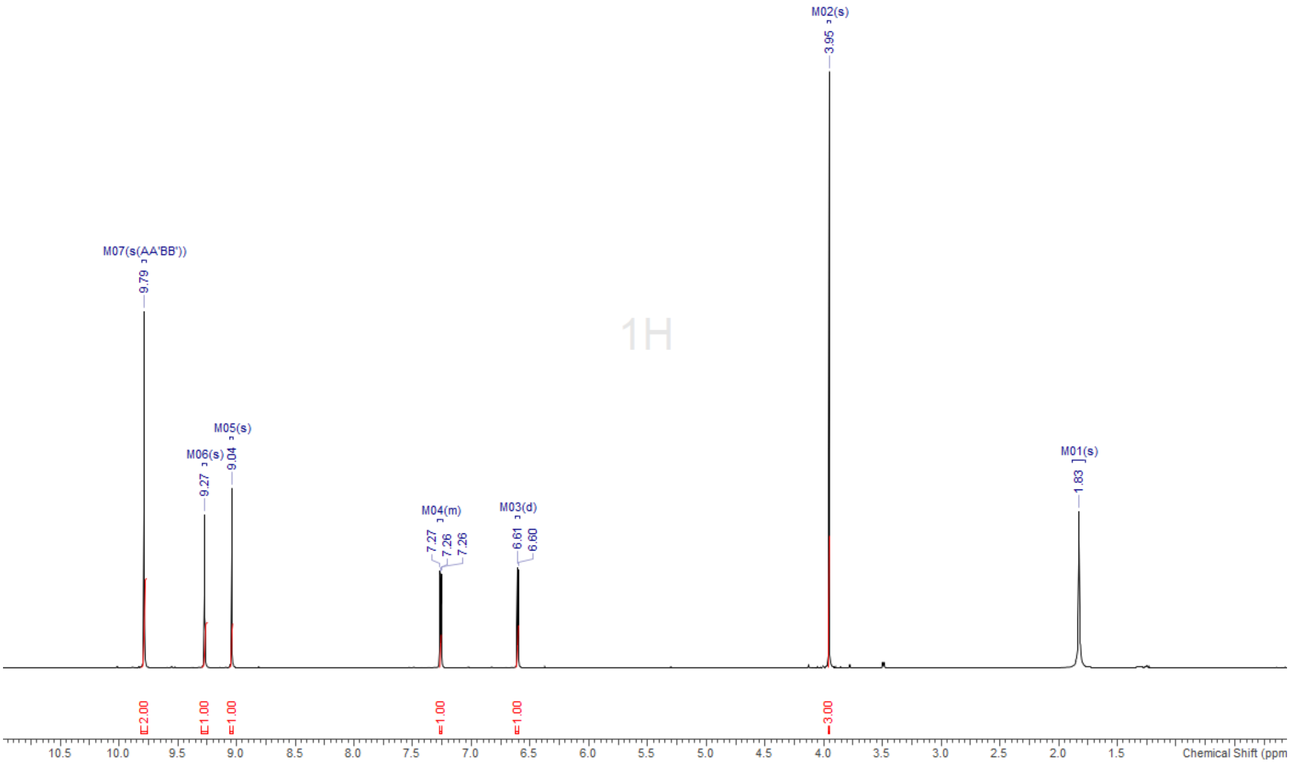

Supplementary Figure 22: Pd-catalysis: ^{1}H NMR (400 MHz, $CDCl_3$) of 7-methyl-2-(5-pyrimidyl)pyrrolo[2,3-d]pyrimidine

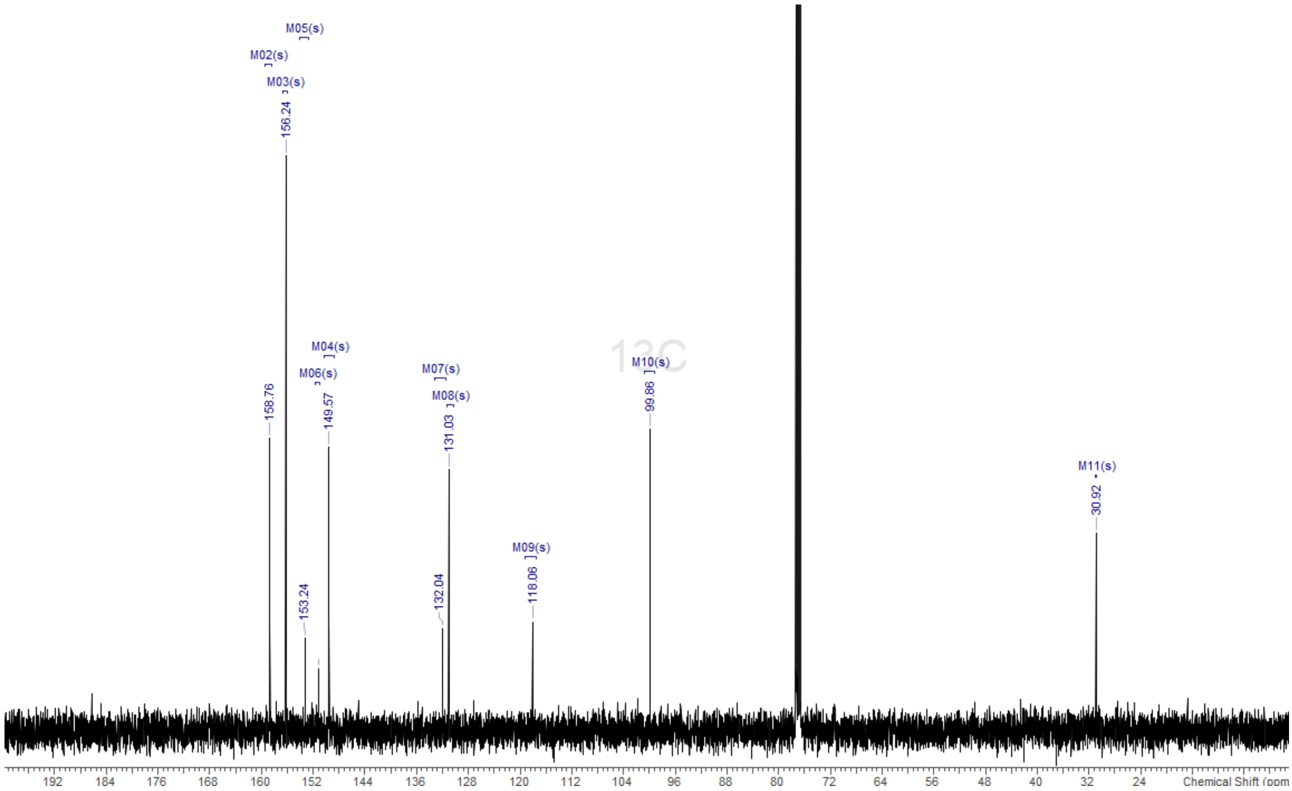

Supplementary Figure 23: Pd-catalysis: $^{13}C\{^{1}H\}$ NMR (101 MHz, $CDCl_3$) of 7-methyl-2-(5-pyrimidyl)pyrrolo[2,3-d]pyrimidine

5.2.6 Visualisation of experimental results

For the ML-driven optimisation of the nickel catalysed Suzuki reaction, we conducted a total of six 96-well HTE plates, encompassing 576 reactions. This was in addition to the 192 experiments conducted with the two chemist-designed HTE batches. Our ML-driven Bayesian optimisation workflow was structured as follows: In iteration 1, we initialised the first plate using quasi-random Sobol sampling. In iterations 2-4, we followed with three iterations of Bayesian optimisation, as detailed in the Application to Nickel-catalysed Suzuki reactions section in the main text. In iteration 5, the final iteration, we explored two different acquisition function strategies concurrently, each implemented on a separate HTE plate, 5a and 5b. In Plate 5a, we continued with the default Bayesian optimisation approach used in iterations 2-4. In Plate 5b, we used a fully exploitative Bayesian optimisation approach using Utopia point scalarisation [7]. This approach aims to maximise the use of all accumulated data in iterations 1-4, focusing entirely on exploitation and neglecting further exploration. We include in this section scatter plots to visually compare the performance of the two parallel strategies explored for the final fifth iteration (Supplementary Figure 24). All HTE reaction data are included in the Simple User-Friendly Reaction Format (SURF) [8] in the GitHub repository accompanying this manuscript.

5.2.7 Data analysis of experimental results

To support empirical observations from all 576 experiments in the ML HTE campaign and further elucidate underlying chemical relationships governing the nickel-catalysed Suzuki reaction, we employed several analysis and visualisation methods. We include in this section box plots comparing the average experimental AP yield value of all ligands (Supplementary Figure 25 and 26), solvents, bases, precatalysts, cosolvents, and temperature (Supplementary Figure 27) to the overall average AP yield

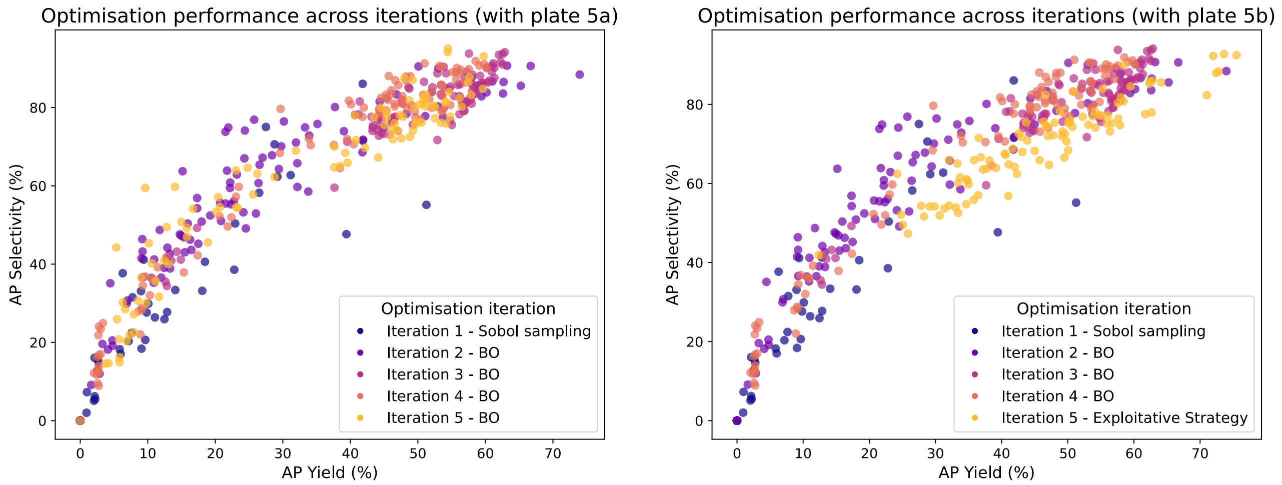

Supplementary Figure 24: Comparison of concurrent optimisation strategies in the fifth and final iteration of the ML optimisation campaign.

of the entire dataset.

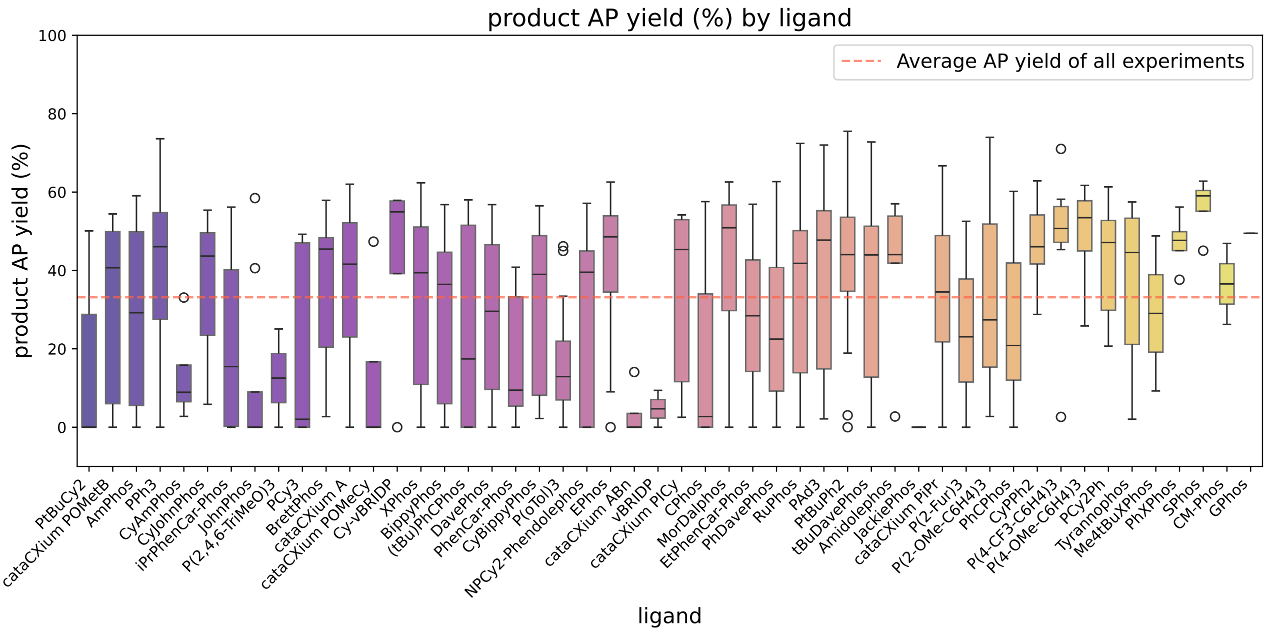

Supplementary Figure 25: Box plot of product AP yield (%) by ligands used in the experimental case study.

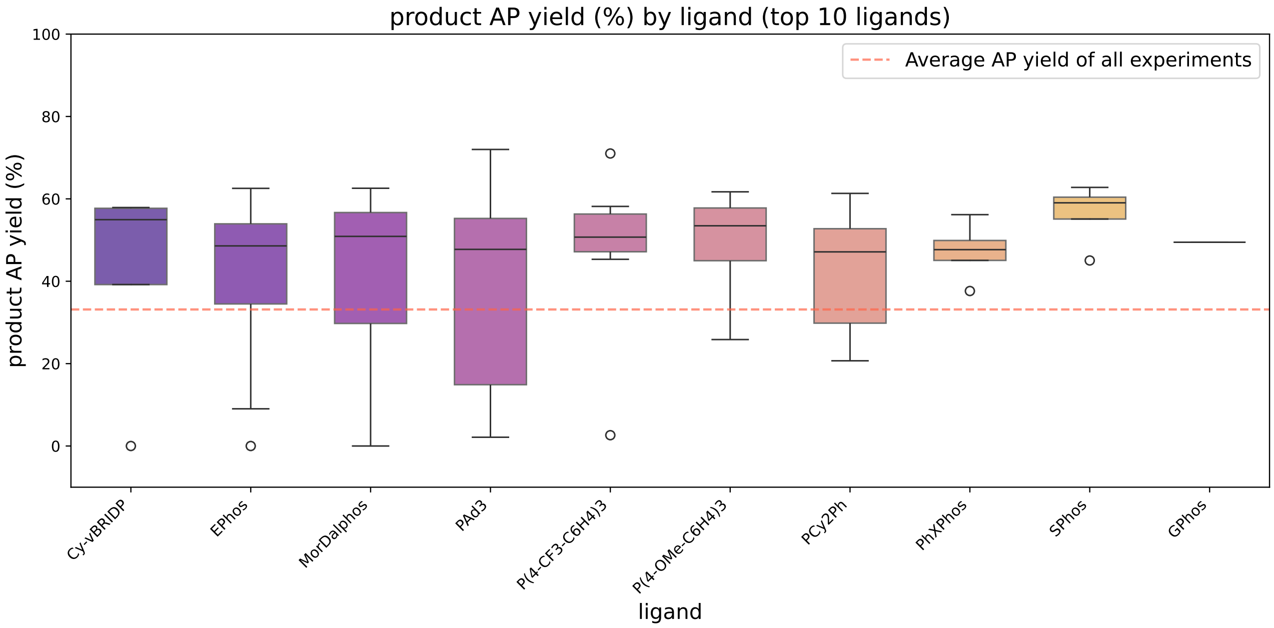

Supplementary Figure 26: Box plot of product AP yield (%) by ligands used in the experimental case study, including only top 10 ligands ranked by median product AP yield (%)

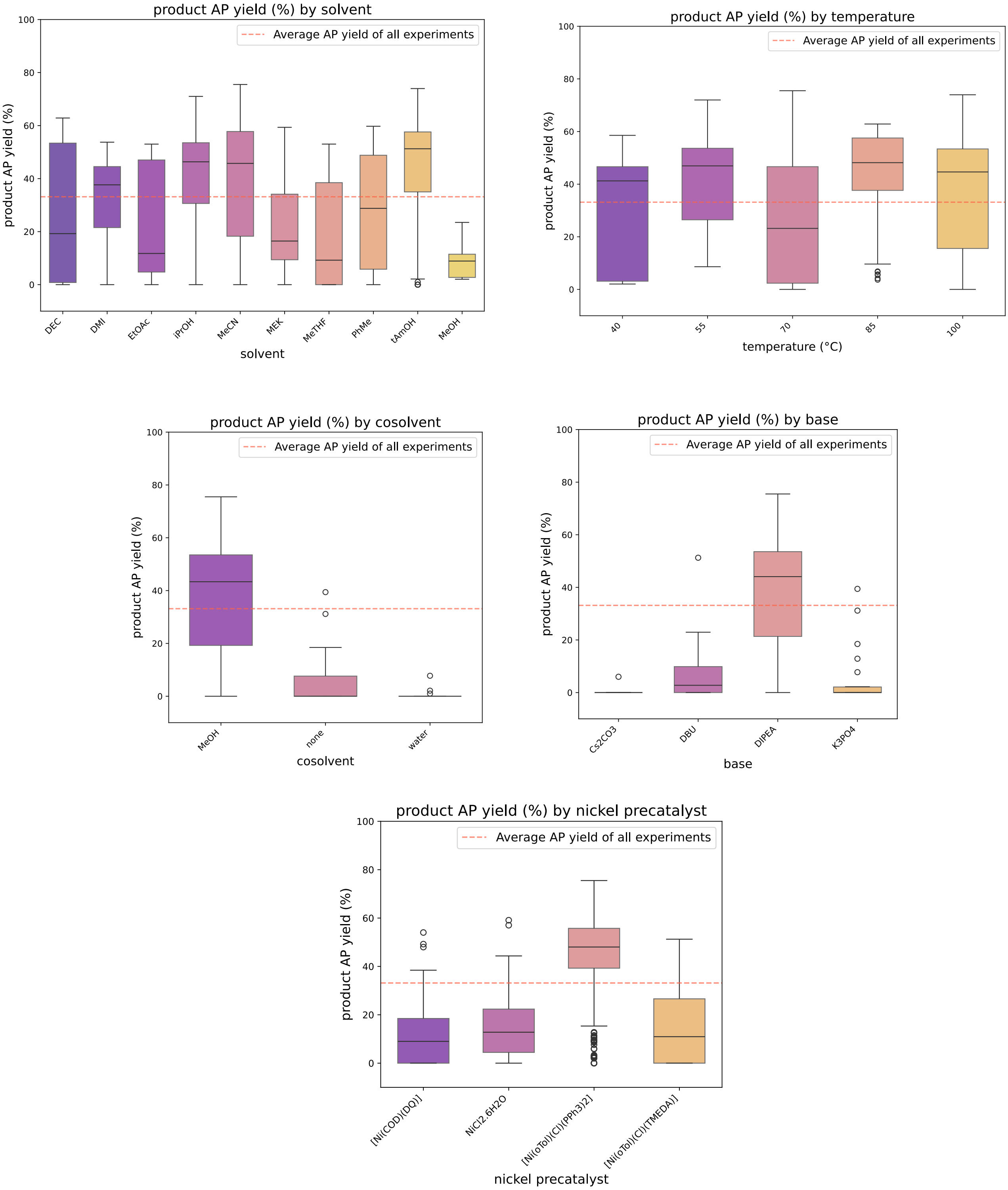

Supplementary Figure 27: Box plot of product AP yield (%) by solvents, temperatures, cosolvents, bases, and nickel precatalysts used in the experimental case study.

5.3 Nickel-catalysed Suzuki-Miyaura coupling: Active pharmaceutical ingredient (API) case study

5.3.1 Reaction condition search space

The reaction parameters and reaction condition search space for the API Ni-catalysed Suzuki reaction were identical to those in the prior case study (Supplementary Figure 16). The combinatorial set of reaction parameter combinations encompassed 120,000 possible configurations, and after removing configurations for which the reaction temperature exceeded the solvent boiling point, we obtained 88,000 possible reaction conditions.

5.3.2 Experimental procedure for HTE campaign with ML optimisation workflow

Solid ligands (3.75 µmol, 5.0 mol%), nickel precursor (1.87 µmol, 2.5 mol%), solid bases (150 µmol, 2.0 eq) and ArBr (75.0 µmol) were dispensed into 1 mL vials with stirring disks in a 96 well plate. Liquid ligands (3.75 µmol, 5.0 mol%), RBpin (113 µmol, 1.5 eq), solvents (180 µL), co-solvents (60 µL), and liquid bases (150 µmol, 2.0 eq) were added and the vials separated into two plates based on the reaction temperatures. The plates were sealed and stirred at the indicated temperature for 18 h. The plates were then recombined, 4/1 MeCN/H_2O (500 µL) was added and shaken for 20 min at rt. 25 µL samples taken and analysed by Liquid Chromatography-Mass Spectroscopy (LC-MS) to obtain area percent (AP) metrics.

5.3.3 Scale-up of HTE results

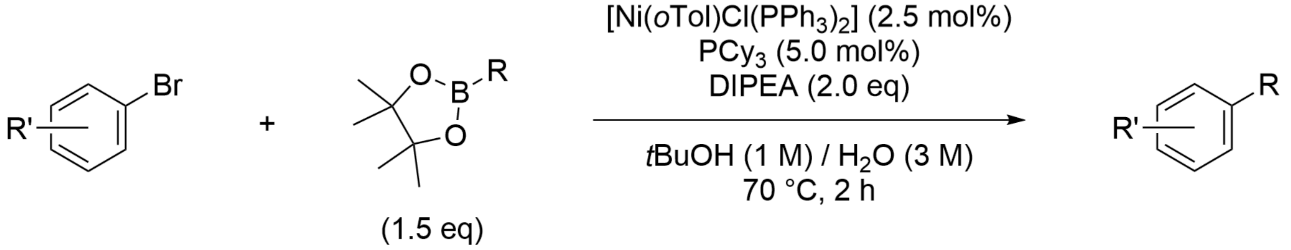

[Ni(oTol)Cl(PPh_3)$_2$] (710 mg, 1.00 mmol, 2.5 mol%) and PCy_3 (561 mg, 2.00 mmol, 5.0 mol%) were stirred in tBuOH (20 mL) for 30 min and the suspension was then added to ArBr (40.0 mmol) and RBpin (60.0 mmol, 1.5 eq) in tBuOH (25 mL). DIPEA (13.9 mL, 80.0 mmol, 2.0 eq) and H_2O (13.3 mL) were added and the mixture was stirred at 70 °C for 2 h. The reaction was cooled to rt, diluted with EtOAc (40 mL) and washed with water (40 mL, re-extracted with EtOAc (40 mL)), 5% aq. HCl solution (40 mL) and sat. aq. $NaHCO_3$ solution (40 mL). The organic phase was dried over Na_2SO_4, filtered and the solvent was removed under reduced pressure. Purification by distillation afforded the product in 86% yield.

5.4 Palladium-catalysed Buchwald-Hartwig coupling: API case study

5.4.1 Reaction condition search space

We include a full list of reaction parameters in the reaction condition search space for the API Pd-catalysed Buchwald-Hartwig coupling (Supplementary Figure 28). The combinatorial set of reaction parameter combinations encompassed 39,600 possible reaction conditions.

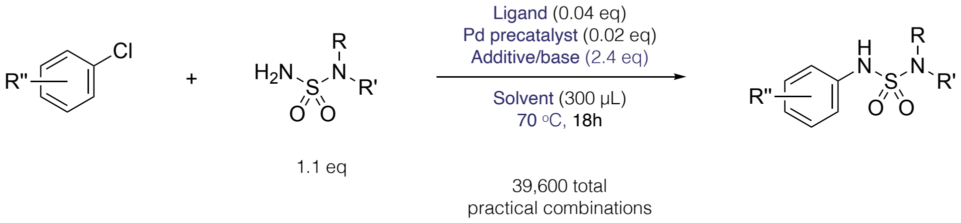

Monophosphine ligand (80)

PPh_3	MeDalPhos	EPhos	Et-PhenCar-Phos
PCy_3	MorDalphos	p-DavePhos	GPhos
$P(tBu)_3$	Me_4tBuXPhos	PhenCar-Phos	Tyrannophos
JohnPhos	JackiePhos	RuPhos-Hybrid	CM-Phos
$P(oTol)_3$	BrettPhos	CPhos	PCy_2Ph
cataCXium ABn	SPhos	PhCPhos	PPh_2-Andolephos
cataCXium A	PhDavePhos	(tBu)PhCPhos	$NPCy_2$-Phendolephos
cataCXium POMetB	tBuBrettPhos	PAd_3	Amidolephos
cataCXium POMeCy	CyAmphos	CPhos-Hybrid	$NPCy_2$-Andolephos
cataCXium PIntB	Amphos	tBuXPhos	Metamorphos
cataCXium PtB	RockPhos	VPhos	Pta
cataCXium PInCy	AdBippyPhos	MePhos	Ph-PhenCar-Phos
cataCXium PCy	BippyPhos	TrixiePhos	$PtBuPh_2$
vBRIDP	CyBippyPhos	TRIPPYPHOS	(rac)-QUINAP
Cy-vBRIDP	RuPhos	PhSPhos	CyRockPhos
tBuDavePhos	AdBrettPhos	tBuSPhos	PAd_2Cy
tBu_2P(Mebiphenyl)	$CyPPh_2$	DMPP	iPr-PhenCarPhos
CyJohnPhos	DavePhos	cataCXium PiPr	PhXPhos
cataCXium PICy	AlPhos	Triisobutylphosphatrane	P(3,5-CF_3-Ph)$_3$
XPhos	(pDMA-Ph)P(Ph)$_2$	(rac)-BI-DIME	VincePhos

Palladium precatalyst (3)

- $[Pd(allyl)Cl]_2$
- $[Pd_2(dba)_3]$
- $[Pd(OAc)_2]$

Solvents (15)

- acetonitrile
- anisole
- p-xylene
- propionitrile
- mestitylene
- toluene
- n-propyl acetate
- tetrahydrofuran
- isobutyl acetate
- di-isopropyl ether
- isoamyl acetate
- di-n-butyl ether
- cyclopentyl-methyl-ether
- 2-methyl-2-butanol
- o-xylene

Base (4)

- KOAc
- NaOAc
- DBU
- Tetramethylguanidine
- diisopropylethyl amine
- KOPivalate
- K_3PO_4
- K_3PO_4/DBU
- KOAc/DBU
- KOPivalate/DBU
- K_3PO_4/KOAc

Supplementary Figure 28: Full reaction condition space for the Pd-catalysed Buchwald-Hartwig API experimental case study using our ML optimisation workflow.

5.4.2 Experimental procedure for HTE campaign with ML optimisation workflow

Solid ligands (1.82 µmol, 4.0 mol%), palladium precursor (0.91 µmol, 2.0 mol% Pd, 10 w% on ChemBeads), solid bases (2.4 eq, or 1.2 eq for dual-base reactions), ArCl (45.5 µmol) and H_2NSO_2NRR' (50.0 µmol, 1.1 eq) were dispensed into 1 mL vials with stirring disks in a 96 well plate. Liquid ligands, solvents (300 µL), and liquid bases (2.4 eq, or 1.2 eq for dual-base reactions) were added. The plate was sealed and stirred at 70 °C for 18 h. 4/1 MeCN/H_2O (500 µL) was added and shaken for 20 min at rt. 50 µL samples taken and analysed by Liquid Chromatography-Mass Spectroscopy (LC-MS) to obtain area percent (AP) metrics.

5.4.3 Scale-up of HTE results

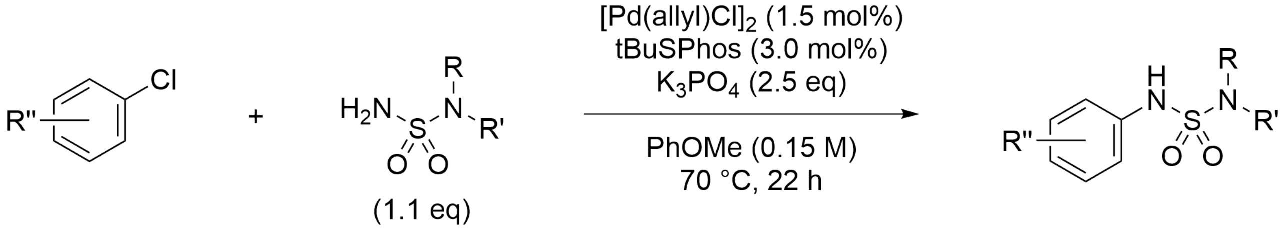

tBuSPhos (32.5 mg, 91.0 µmol, 3.0 mol%) and $[Pd(allyl)Cl]_2$ (16.7 mg, 45.5 µmol, 1.5 mol%) in PhOMe (2.0 mL) were added to ArCl (3.03 mmol), H_2NSO_2NRR' (3.34 mmol, 1.1 eq) and K_3PO_4 (1.61 g, 7.58 mmol, 2.5 eq) in PhOMe (18 mL) and the mixture was stirred at 70 °C overnight. HPLC analysis showed >99% conversion and <0.5% of the undesired side product.

References

[1] Jose Antonio Garrido Torres, Sii Hong Lau, Pranay Anchuri, Jason M. Stevens, Jose E. Tabora, Jun Li, Alina Borovika, Ryan P. Adams, and Abigail G. Doyle. A multi-objective active learning platform and web app for reaction optimization. *Journal of the American Chemical Society*, 144:19999–20007, 11 2022.

[2] Florian Häse, Matteo Aldeghi, Riley J Hickman, Loïc M Roch, Melodie Christensen, Elena Liles, Jason E Hein, and Alán Aspuru-Guzik. Olympus: a benchmarking framework for noisy optimization and experiment planning. *Machine Learning: Science and Technology*, 2:035021, 9 2021.

[3] Andreia P. Guerreiro, Carlos M. Fonseca, and Luís Paquete. The hypervolume indicator: Problems and algorithms, 2020.

[4] R. F. Woolson. Wilcoxon signed-rank test, September 2008.

[5] Adam Paszke, Sam Gross, Francisco Massa, Adam Lerer, James Bradbury, Gregory Chanan, Trevor Killeen, Zeming Lin, Natalia Gimelshein, Luca Antiga, Alban Desmaison, Andreas Köpf, Edward Yang, Zach DeVito, Martin Raison, Alykhan Tejani, Sasank Chilamkurthy, Benoit Steiner, Lu Fang, Junjie Bai, and Soumith Chintala. Pytorch: An imperative style, high-performance deep learning library, 2019.

[6] Georg Wuitschik, Vera Jost, Torsten Schindler, and Michal Jakubik. Hte os: A high-throughput experimentation workflow built from the ground up. *Organic Process Research & Development*, 28(7):2875–2884, 2024.

[7] Derek M. Dalton, Richard C. Walroth, Caroline Rouget-Virbel, Kyle A. Mack, and F. Dean Toste. Utopia point bayesian optimization finds condition-dependent selectivity for n-methyl pyrazole condensation. *Journal of the American Chemical Society*, 146(23):15779–15786, May 2024.

[8] David F. Nippa, Alex T. Müller, Kenneth Atz, David B. Konrad, Uwe Grether, Rainer E. Martin, and Gisbert Schneider. Simple user-friendly reaction format, May 2024.
