# Supplementary material for: Highly parallel optimisation of chemical reactions through automation and machine intelligence
Source: Nat Commun. 2025 Jul 12;16:6464. doi: 10.1038/s41467-025-61803-0 (PMC12255721; doi:10.1038/s41467-025-61803-0)
Supplement: Supplementary file 2 — Reporting Summary [file 41467_2025_61803_MOESM2_ESM.pdf]

nature portfolio

Corresponding author(s): Joshua W. Sin, Philippe Schwaller

Last updated by author(s): May 18, 2025

Reporting Summary

Nature Portfolio wishes to improve the reproducibility of the work that we publish. This form provides structure for consistency and transparency in reporting. For further information on Nature Portfolio policies, see our Editorial Policies and the Editorial Policy Checklist.

Statistics

For all statistical analyses, confirm that the following items are present in the figure legend, table legend, main text, or Methods section.

n/a	Confirmed	
☐	☒	The exact sample size (*n*) for each experimental group/condition, given as a discrete number and unit of measurement
☒	☐	A statement on whether measurements were taken from distinct samples or whether the same sample was measured repeatedly
☐	☒	The statistical test(s) used AND whether they are one- or two-sided<br>*Only common tests should be described solely by name; describe more complex techniques in the Methods section.*
☒	☐	A description of all covariates tested
☒	☐	A description of any assumptions or corrections, such as tests of normality and adjustment for multiple comparisons
☒	☐	A full description of the statistical parameters including central tendency (e.g. means) or other basic estimates (e.g. regression coefficient) AND variation (e.g. standard deviation) or associated estimates of uncertainty (e.g. confidence intervals)
☒	☐	For null hypothesis testing, the test statistic (e.g. *F*, *t*, *r*) with confidence intervals, effect sizes, degrees of freedom and *P* value noted<br>*Give P values as exact values whenever suitable.*
☒	☐	For Bayesian analysis, information on the choice of priors and Markov chain Monte Carlo settings
☒	☐	For hierarchical and complex designs, identification of the appropriate level for tests and full reporting of outcomes
☒	☐	Estimates of effect sizes (e.g. Cohen's *d*, Pearson's *r*), indicating how they were calculated

Our web collection on statistics for biologists contains articles on many of the points above.

Software and code

Policy information about availability of computer code

Data collection

The custom code software (Minerva v1.0.0) used to analyse data and collect results is deposited as an open source code repository on Zenodo:
https://doi.org/10.5281/zenodo.15455502

Minerva was built using PyTorch (2.0.1), BoTorch (0.6.0), and GPyTorch (1.6.0). All plots were generated with Matplotlib (3.7.4).

The other packages used for data collection and analysis are specified below:
pykeops==1.5
pytorch-lightning==2.1.2
scikit-learn==1.3.2
scipy==1.10.1
shap==0.44.1
tensorflow==2.13.1

Data analysis

The custom code software (Minerva v1.0.0) used to analyse data and collect results is deposited as an open source code repository on Zenodo:
https://doi.org/10.5281/zenodo.15455502

Minerva was built using PyTorch (2.0.1), BoTorch (0.6.0), and GPyTorch (1.6.0). All plots were generated with Matplotlib (3.7.4).

The other packages used for data collection and analysis are specified below:
pykeops==1.5
pytorch-lightning==2.1.2
scikit-learn==1.3.2
scipy==1.10.1
shap==0.44.1
tensorflow==2.13.1

For manuscripts utilizing custom algorithms or software that are central to the research but not yet described in published literature, software must be made available to editors and reviewers. We strongly encourage code deposition in a community repository (e.g. GitHub). See the Nature Portfolio guidelines for submitting code & software for further information.

Data

Policy information about availability of data

All manuscripts must include a data availability statement. This statement should provide the following information, where applicable:

- Accession codes, unique identifiers, or web links for publicly available datasets
- A description of any restrictions on data availability
- For clinical datasets or third party data, please ensure that the statement adheres to our policy

The data generated in this study is deposited in an open source repository on Zenodo: doi.org/10.5281/zenodo.15455502
The data is also available in an open source GitHub repository: github.com/schwallergroup/minerva

Research involving human participants, their data, or biological material

Policy information about studies with human participants or human data. See also policy information about sex, gender (identity/presentation), and sexual orientation and race, ethnicity and racism.

Reporting on sex and gender	N/A
Reporting on race, ethnicity, or other socially relevant groupings	N/A
Population characteristics	N/A
Recruitment	N/A
Ethics oversight	N/A

Note that full information on the approval of the study protocol must also be provided in the manuscript.

Field-specific reporting

Please select the one below that is the best fit for your research. If you are not sure, read the appropriate sections before making your selection.

☒ Life sciences ☐ Behavioural & social sciences ☐ Ecological, evolutionary & environmental sciences

For a reference copy of the document with all sections, see nature.com/documents/nr-reporting-summary-flat.pdf

Life sciences study design

All studies must disclose on these points even when the disclosure is negative.

Sample size	For our computational experiments, we evaluated optimization performance of each ML strategy using 20 independent runs with different random seeds to ensure robust assessment of algorithm performance across different random states. This number of runs is consistent with standard practice in machine learning literature for evaluating Bayesian optimization algorithms. The number of iterations in our benchmarks was determined by monitoring when the mean hypervolume percentage converged to its maximum value, indicating that the algorithm had effectively identified the optimal reaction conditions.<br><br>For our experimental high-throughput experimentation (HTE) campaigns, we conducted 4-5 iterations of 96-well plates (384-480 total reactions per optimization) based on practical considerations of pharmaceutical process timelines and empirical convergence patterns observed in our computational benchmarks. Importantly, these optimization campaigns were terminated when objectives reached theoretical maxima (e.g., >95% yield and >95% selectivity) or when additional iterations showed no further improvement.
Data exclusions	No data were excluded from analyses in this study. All generated experimental results and observations were included in our analysis and reporting, with no pre-established or post-hoc exclusion criteria applied.
Replication	The identified high-performing reaction conditions from our ML-guided high-throughput experimentation campaigns were replicated and verified at gram scale, confirming the validity of the reaction condition hit.

	Furthermore, our machine learning framework was specifically designed with noise robustness in mind. We demonstrate in this study that its performance remains robust even when substantial amounts of simulated experimental noise are introduced.<br><br>In addition, our experimental work was conducted using automated equipment with standardized reaction execution protocols, which reduces experimental variability and enhances reproducibility across runs
Randomization	Our study does not involve traditional experimental groups in the biological/medical sense.<br><br>Each chemical transformation we assess in our study (e.g., Ni-catalyzed Suzuki coupling and Pd-catalyzed Buchwald-Hartwig coupling) represents a separate optimization campaign with its own distinct reaction space.<br><br>Analysis was performed within each reaction campaign independently, comparing optimisation objectives (e.g., AP yield, AP selectivity, Catalyst turnover) across different reaction conditions within the same chemical transformation. We did not compare or analyze optimisation objectives across different reaction types, as each represents a distinct chemical system with its own optimization objectives and parameters.
Blinding	N/A

Reporting for specific materials, systems and methods

We require information from authors about some types of materials, experimental systems and methods used in many studies. Here, indicate whether each material, system or method listed is relevant to your study. If you are not sure if a list item applies to your research, read the appropriate section before selecting a response.

Materials & experimental systems

n/a	Involved in the study
☒	☐ Antibodies
☒	☐ Eukaryotic cell lines
☒	☐ Palaeontology and archaeology
☒	☐ Animals and other organisms
☒	☐ Clinical data
☒	☐ Dual use research of concern
☒	☐ Plants

Methods

n/a	Involved in the study
☒	☐ ChIP-seq
☒	☐ Flow cytometry
☒	☐ MRI-based neuroimaging

Plants

Seed stocks	N/A
Novel plant genotypes	N/A
Authentication	N/A
